# Molecular and Cellular Adaptations in Hippocampal Parvalbumin Neurons Mediate Behavioral Responses to Chronic Social Stress

**DOI:** 10.3389/fnmol.2022.898851

**Published:** 2022-06-24

**Authors:** Dionnet L. Bhatti, Lucian Medrihan, Michelle X. Chen, Junghee Jin, Kathryn A. McCabe, Wei Wang, Estefania P. Azevedo, Jose H. Ledo, Yong Kim

**Affiliations:** ^1^Laboratory of Molecular and Cellular Neuroscience, The Rockefeller University, New York, NY, United States; ^2^Program in Neuroscience, Harvard Medical School, Boston, MA, United States; ^3^Bioinformatics Resource Center, The Rockefeller University, New York, NY, United States; ^4^Laboratory of Molecular Genetics, The Rockefeller University, New York, NY, United States; ^5^Department of Neurosurgery, Robert Wood Johnson Medical School, Rutgers University, Piscataway, NJ, United States; ^6^Brain Health Institute, Rutgers University, Piscataway, NJ, United States

**Keywords:** chronic stress, susceptibility, resilience, dentate gyrus, parvalbumin interneurons, Ahnak

## Abstract

Parvalbumin-expressing interneurons (PV neurons) maintain inhibitory control of local circuits implicated in behavioral responses to environmental stressors. However, the roles of molecular and cellular adaptations in PV neurons in stress susceptibility or resilience have not been clearly established. Here, we show behavioral outcomes of chronic social defeat stress (CSDS) are mediated by differential neuronal activity and gene expression in hippocampal PV neurons in mice. Using *in vivo* electrophysiology and chemogenetics, we find increased PV neuronal activity in the ventral dentate gyrus is required and sufficient for behavioral susceptibility to CSDS. PV neuron-selective translational profiling indicates mitochondrial oxidative phosphorylation is the most significantly altered pathway in stress-susceptible versus resilient mice. Among differentially expressed genes associated with stress-susceptibility and resilience, we find Ahnak, an endogenous regulator of L-type calcium channels which are implicated in the regulation of mitochondrial function and gene expression. Notably, Ahnak deletion in PV neurons impedes behavioral susceptibility to CSDS. Altogether, these findings indicate behavioral effects of chronic stress can be controlled by selective modulation of PV neuronal activity or a regulator of L-type calcium signaling in PV neurons.

## Introduction

Stress enhances motivational drives that promote evolutionarily favorable behaviors critical for survival (Hollon et al., 2015). However, stressful life events or chronic exposure to unavoidable stress can lead to maladaptive cellular and behavioral responses and contribute to the etiology of neuropsychiatric disease such as major depressive disorder (MDD) (Kessler, 1997; Kendler et al., 1999; Nestler et al., 2002; McEwen, 2012; Syed and Nemeroff, 2017). Many stress-related psychiatric disorders are characterized by altered behavioral and physiological states detrimental to an individual. However, while stress exposure can render some individuals susceptible to developing MDD, others are resilient and often remain healthy (Southwick et al., 2005). Several animal models including “learned helplessness,” chronic unpredictable stress, restraint stress and chronic social defeat stress (CSDS) have been used for the studies of stress susceptibility and resilience (Cryan and Mombereau, 2004; Krishnan et al., 2007; Nasca et al., 2015). Among them, the CSDS paradigm has been widely used and led to studies that attribute this individual divergence to molecular and cellular adaptations in multiple brain regions (Han and Nestler, 2017; Cathomas et al., 2019). Nonetheless, the key molecular and cellular targets responsible for behavioral responses to stress are not fully understood.

Alterations of ventral hippocampus are highly implicated in stress and emotional responses (Campbell and Macqueen, 2004; Fanselow and Dong, 2010). Particularly, multiple neuronal types including granule cells, mossy cells and interneurons in the dentate gyrus (DG) have been studied in regard to depression-like behavior and antidepressant action (Malberg et al., 2000; Oh et al., 2013; Shuto et al., 2020; Umschweif et al., 2021a). Previously, we found that molecular alterations selectively in parvalbumin (PV)-expressing GABAergic interneurons could modulate depression-like behavior (Lee et al., 2015; Jin et al., 2020). However, whether adaptations in neuronal firing and molecular expression in PV neurons mediate individual differences in stress vulnerability has not been elucidated.

In this study, we find that susceptibility to CSDS is driven by increased PV neuronal activity in the ventral dentate gyrus (PV*^vDG^*). Furthermore, PV neuron-selective RNA sequencing revealed that CSDS alters multiple molecular pathways regulating mitochondrial function, protein synthesis and synaptogenesis, which could underlie differences in neuronal activity. Among the dysregulated transcripts in hippocampal PV neurons was mRNA for Ahnak. Previously, Ahnak was characterized as an endogenous regulator of L-type voltage-gated calcium channels in PV neurons (Jin et al., 2020), cardiomyocytes (Haase et al., 2005) and T cells (Matza et al., 2008). L-type calcium signaling is highly implicated in mitochondrial function (Guzman et al., 2018; Hotka et al., 2020), gene expression (Dolmetsch et al., 2001; D’Arco and Dolphin, 2012) and neuronal plasticity (Simms and Zamponi, 2014). Importantly, Ahnak expression in the ventral dentate gyrus and in PV neurons is also required for behavioral susceptibility. Together, this study reveals that molecular and cellular adaptations in hippocampal PV neurons incurred by social stress mediate behavioral susceptibility or resilience.

## Materials and Methods

### Animals

All experiments involving animals were approved by The Rockefeller University Institutional Animal Care and Use Committee and were in accordance with the National Institutes of Health guidelines. Floxed Ahnak mice were generated and maintained at The Rockefeller University as described previously (Jin et al., 2020). Floxed Ahnak mice were crossed with PV-Cre mice (stock no: 008069, The Jackson Laboratory) to generate PV neuron-selective Ahnak KO line. Cre-dependent EGFP-L10a mice (stock no: 024750, The Jackson Laboratory, Bar Harbor, ME, United States) were crossed with PV-Cre mice to generate PV neuron-selective EGFP-L10a line for translating ribosomal affinity purification (TRAP). We produced the progeny of floxed Ahnak, PV-Cre, PV-selective EGFP-L10a and PV-selective Ahnak KO lines by *in vitro* fertilization (IVF) and embryo transfer techniques (Transgenic and Reproductive Technology Center, The Rockefeller University) to provide genotype- and age-matched animals. All mice are male and of C57BL/6 background except CD1 aggressors (strain 022, Charles River, Kingston, NY, United States) used for CSDS. Mice were housed 3–5 per cage with a 12:12-h light/dark cycle and *ad lib* access to food and water. *In vivo* recordings were performed with anesthetized mice. All behavioral tests were performed during the light cycle. All behavioral experiments commenced with male mice aged 8–12 weeks old. Transgenic mice were assigned randomly to experimental stress conditions based on their genotype. For AAV-mediated gene delivery experiments, transgenic mice were randomly assigned to experimental virus groups and then later to experimental stress conditions. Experimenters were blinded to group or genotype when performing and analyzing western blots, RNAscope, and social defeat. Experimenters were not blinded to groups when conducting behavioral experiments, but mice from each group were evenly assigned to equipment, run in parallel during behavioral tests, and analyzed using automated tracking software (Ethovision).

### Stereotaxic Surgery

All stereotaxic surgeries were performed on an Angle Two Small Animal Stereotaxic Instrument (Leica Biosystems, Buffalo Grove, IL, United States) with a microinjection syringe pump (UMP3 UltraMicroPump, World Precision Instruments, Sarasota, FL, United States). Male mice (7–8 weeks of age) were anesthetized with a mix of ketamine (100 mg/ml) and xylazine (10 mg/ml). AAV5-hSyn-GFP and AAV5-hSyn-Cre-GFP were obtained from UNC Vector Core, and AAV5-hSyn-DIO-mCherry (50459), AAV5-hSyn-DIO-hM4D(Gi)-mCherry (44362) and AAV5-hSyn-DIO-hM3D(Gq)-mCherry (44361) were purchased from Addgene. Viruses (500 nl/side) were injected bilaterally into the vDG (AP −2.7 ML ± 2.0 DV −2.2, mm relative to bregma) with a 2 mL Hamilton Neuros syringe at a speed of 0.1 μl/min. The needle was left for an additional 10 min and then slowly withdrawn. The stereotaxic injections were confirmed by immunohistochemistry and animals with infection outside of vDG or no infection in the vDG were excluded from data analysis. Mice were monitored for 48 h to ensure full recovery from the surgery. Experiments commenced 3 weeks after stereotaxic surgery to allow optimal expression of AAV viruses. Previous studies indicate that a single low dose of ketamine has a long-lasting antidepressant-like effects up to 8 days in rodents (Autry et al., 2011; Ma et al., 2013; Kim et al., 2021). In this study, we used ketamine as anesthesia for stereotaxic surgeries and AAV-mediated gene delivery. Because we performed experiments 3 weeks after stereotaxic surgery, we believe any potential effect of ketamine had waned down before the commencing the experiments. Importantly, all control groups in Figure 2 and Figures 5A–E underwent the same anesthetic procedure and stereotaxic injection of control AAV, suggesting that the outcome we observed are due to DREADD (designer receptor exclusively activated by designer drugs)-mediated inhibition or activation (Figure 2) or Ahnak KO and not influenced by ketamine.

### Chronic Social Defeat Stress

Chronic social defeat stress (CSDS) was carried out as described previously (Golden et al., 2011). Retired male breeder CD-1 mice were screened over three consecutive days and aggressors were selected according to the following criteria: (i) the latency to the initial attack was under 60 s and (ii) the screener mouse was attacked for two consecutive days. For ten consecutive days, the experimental mice were placed in the home cage of a prescreened CD-1 aggressor for 5-min of physical attack and then separated by a perforated divider for the remaining 24 h until the next defeat 24 h later. Each experimental mouse was exposed to a different aggressor each day. In parallel, stress-naïve control mice were placed in pairs within an identical home cage setup separated by a perforated divider for the duration of the defeat sessions. They were never in physical or sensory contact with CD-1 mice. After 10 days of social defeat, all aggressors and experimental mice were separated and singly housed. The SI test was performed 24 h later.

### Subthreshold Social Defeat Stress

Subthreshold social defeat stress (SSDS) was carried out as described previously (Cheng et al., 2019). Experimental mice were placed in the home cage of a prescreened CD-1 aggressor for 5-min of physical attack. 15 min later, the experimental mice were introduced to another 5-min physical attack by a novel CD-1 aggressor. This was repeated once more for a total of three defeat sessions. The SI test was performed 24 h later.

### Social Interaction Test

Social interaction (SI) test was carried out as described previously (Golden et al., 2011). The test was composed of two phases, each consisting of 150 s, where the experimental mice were allowed to explore an open field (42 cm × 42 cm × 42 cm) with a wire mesh enclosure (10 cm wide × 6.5 cm deep × 42 cm high). In the first phase, the wire mesh enclosure was empty. In the second phase, a novel CD-1 aggressor mouse was placed inside the wire mesh. The amount of time the experimental mice spent in the interaction zone surrounding the wire mesh enclosure was collected and analyzed by the video-tracking apparatus and software EthoVision XT 7 (Noldus Information Technology, Leesburg, VA, United States). SI ratio was calculated by dividing the amount of time the experimental mice spent in the interaction zone in phase two by the time in phase one [SI Ratio = (phase 2 time)/(phase 1 time)]. Susceptible mice were defined by a SI ratio under 1, whereas resilient mice were defined by a SI ratio greater than 1 (Golden et al., 2011).

### Sucrose Preference Test

Sucrose Preference Test (SPT) was adapted from previous studies (Jin et al., 2020). During the 1-day habituation period, mice were given a choice of two water bottles. The following day, bottles were replaced with new bottles containing either water or 2% sucrose solution. The consumption of water and sucrose solution was measured at different timepoints (0 and 4 h for acute CNO experiment or 24 h for chronic CNO and genetic knockout experiments). The sucrose preference was represented as percent preference for sucrose {[sucrose consumed/(water + sucrose consumed)] × 100}.

### Drug Administration

For *in vivo* electrophysiology experiments, susceptible mice were selected after the SI test. Ketamine administration (10 mg/kg, i.p., k2753, Sigma-Aldrich) occurred 24 h after the SI test and 1 day before the *in vivo* recording experiment.

For all Gi and Gq DREADD experiments, CNO (3 mg/kg, i.p., C0832, Sigma-Aldrich) (Medrihan et al., 2017) was administered. For chronic CNO administration in h4MDi animals during CSDS (Figure 2C), CNO was administered 30 min prior to each defeat for all 10 days of defeat. SI test was performed drug-free. For CNO administration in hM4Di animals after CSDS (Figure 2G), CNO was administered 30 min prior to the SI test and SPT. For CNO administration in hM3Dq animals during SSDS (Figure 2L), CNO was administered 30 min prior to the first subthreshold defeat session. SI-1 test was performed drug-free. For chronic CNO administration in h3MDq animals (Figure 2L), CNO was administered once each day for 10 days beginning the day after the acute administration and SI-1 test. SI-2 test was performed drug-free.

### *In vivo* Electrophysiology

Animals were anesthetized by injection of urethane (1 mg/kg, i.p.). For craniotomy, mice were mounted in a stereotaxic frame (David Kopf Instruments, CA, United States), in which the head of the animal was fixed with a pair of ear bars and a perpendicular tooth bar. Body temperature was continuously monitored by a rectal thermometer and maintained at 33 ± 1°C by placing the animal on a heating pad. Measurements were obtained from the ventral hippocampus. Stereotaxic coordinates [in mm, anterioposterior (−2.92) measured from bregma; lateral (±2.00) specified from midline; dorsoventral (−2.20) from surface of the brain] were set according to the Franklin and Paxinos Mouse Brain atlas (3rd edition). High density silicon probes with 4 shanks were used (Buzsaki32, NeuroNexus Inc., MI). The shanks were 250 μm apart from each other and bore eight recording sites each (160 μm diameter for each site; 1–3 MΩ impedance) arranged in a staggered configuration with 20 μm vertical separation. The probes were connected to a RHD 2132 amplifier board with 32 channels (Intan Technologies, CA, United States), mounted on a micromanipulator (Luigs & Neumann, Germany) and were gently inserted in the craniotomy window targeting the granule cell layer of the dentate gyrus in ventral hippocampus (AP –2.9 mm, L 2 mm, DV –2.2 mm). Data were sampled at 20 KHz.

For the spike detection, we employed a fully automated approach with the MountainSort clustering software (publically available at^1^). Spikes were verified using the MountainView software, available in the same package, and the various parameters were imported in Matlab for the spike sorting procedure (Matlab Signal Processing Toolbox, Mathworks, MA, United States). For the spike sorting procedure, two features of the spike were initially computed: trough-to-peak latency and the bursting behavior. Narrow waveform neurons, likely putative parvalbumin-positive basket cells (Stark et al., 2013), were classified as neurons with trough-to-peak smaller than 0.4 ms; wide waveforms were classified as neurons with half peaks longer than 0.4 ms. For the bursting behavior, a bursting index was calculated from the ratio of the frequency distribution of inter-spike intervals at 0-10 ms to that of the frequency distribution of inter-spike intervals at 200–300 ms. Neurons with a bursting index higher than 1.8 were considered excitatory neurons (Senzai and Buzsaki, 2017). To further separate the excitatory neurons, we took advantage of our own previous experience with intracellular recording with mossy cells and granule cells (Toader et al., 2013). The total number of neurons detected in all analyzed animals in the study (*n* = 772) went through a first stage classification based on the through-to-peak latency and burst index (BI). Three divisions were classified: putative excitatory cells (black dots, *n* = 336, BI > 1.8), narrow-waveform (red dots, *n* = 142, latency ≤ 0.4 ms) and wide-waveform (green dots, *n* = 294, latency > 0.4 ms) putative interneurons. Based on the bimodal distribution of the afterhyperpolarization (AHP) current, measured from the first derivative of the spike, excitatory neurons were further classified in putative mossy cells (*n* = 133, AHP < 70) and putative granule cells (*n* = 203, AHP > 70). In patch-clamp experiments, the intracellular action potentials of mossy cells show a significantly smaller AHP when compared to the mossy cells (L.M., unpublished data). To analyze the AHP in our experiments, we used the first derivative of the excitatory spikes (dx = dV/ds) in order to avoid the possible errors induced by the variable distance of the respective cells to the recording electrode. This approach also maximizes the differences in AHP induced by the potentially different dynamics between the ion channel conductances in the two neuronal types. We noticed a bimodal distribution of AHP in the excitatory neurons and according to it, we set a threshold of 70 (μV/ms) as a separation between mossy cells (< 70) and granule cells (> 70).

### Slice Electrophysiology

Four-week-old mice were euthanized with CO_2_. Following decapitation and removal of the brains, transversal slices (400 μm thickness) were cut using a Vibratome 1000 Plus (Leica Microsystems, IL, United States) at 2°C in a cutting solution containing (in mM): 87 NaCl, 25 NaHCO_3_, 2.5 KCl, 0.5 CaCl_2_, 7 MgCl_2_, 25 glucose, 75 sucrose and saturated with 95% O_2_ and 5% CO_2_. After cutting, the slices were left to recover for 30–45 min at 35 °C and then for 1 h at room temperature in recording solution (aCSF). The aCSF solution contained (in mM): 125 NaCl, 25 NaHCO_3_, 2.5 KCl, 1.25 NaH_2_PO_4_, 2 CaCl_2_, 1 MgCl_2_ and 25 glucose (bubbled with 95% O2 and 5% CO2). Whole-cell patch-clamp recordings were performed with a Multiclamp 700B/Digidata1550A system (Molecular Devices, CA, United States) and an upright Olympus BX51WI microscope (Olympus, Japan). An individual slice was placed in a recording chamber (RC-27L, Warner Instruments, United States) and constantly perfused with oxygenated aCSF at 24°C (TC-324B, Warner Instruments, United States) at a rate of 1.5–2.0 ml/min. Whole-cell patch-clamp recordings were obtained from PV neurons identified based on their size, shape and position in the subgranular layer using recording pipettes (Glass type 8250, King Precision Glass, Inc., CA, United States) that were pulled in a horizontal pipette puller (Narishige, NY) to a resistance of 3–4 MΩ and filled with an internal solution containing (in mM): 126 K-gluconate, 4 NaCl, 1 MgSO_4_, 0.02 CaCl_2_, 0.1 BAPTA, 15 glucose, 5 HEPES, 3 ATP, 0.1 GTP (pH 7.3). In order to measure the firing of the PV neurons, steps of 100 pA current were injected from a set starting membrane potential of −70 mV. Properties of the single action potentials were measured from the first action potential induced by the steps of injected current. Data were acquired at a sampling frequency of 50 kHz and filtered at 1 kHz and analyzed offline using pClamp10 software (Molecular Devices, CA, United States).

### TRAP and RNA-seq

TRAP was conducted as previously described (Heiman et al., 2014; Roussarie et al., 2020). Briefly, PV neuron-selective TRAP mice were subjected to CSDS. Hippocampi from PV-TRAP mice were freshly harvested. Hippocampal homogenates of non-defeat, resilient and susceptible mice were used for immunoprecipitation of EGFP-tagged polysomes from PV neurons, and polysome-attached mRNAs were isolated and RNA was further purified using RNeasy Micro Kit (Qiagen, Hilden, Germany). All RNA samples were validated for high quality using Bioanalyzer RNA 6000 Pico Kit (Agilent, San Diego, CA, United States). 1 ng of total RNA was used to generate full length cDNA using Clontech’s SMART-Seq v4 Ultra Low Input RNA Kit. 1 ng of cDNA was then used to prepare libraries using Illumina Nextera XT DNA sample preparation kit. Libraries with unique barcodes were pooled at equal molar ratios and sequenced on Illumina NextSeq 500 sequencer to generate 150 bp single reads, following manufacture’s protocol. Raw data can be found in the Nation Center for Biotechnology Information (NCBI) Gene Expression Omnibbus (GEO) database (GSE184027).

### Bioinformatics Analysis

Following sequencing, adapter and low-quality bases were trimmed by fastp (Chen et al., 2018) from the raw sequencing files in FASTQ format. Cleaned reads were aligned to the Mus musculus assembly 10 reference genome using STAR version 2.7.1a (Dobin et al., 2013). After alignment, the Reads Per Kilobase of transcript per Million mapped reads (RPKM) for all genes in each sample were calculated with R package edgeR (McCarthy et al., 2012). To analyze differential gene expression between samples, DESeq2 (Love et al., 2014) was used, applying the standard comparison mode between two experimental groups. *P* values were calculated in DESeq2 adjusted for multiple testing using the Benjamini-Hochberg procedure. Ingenuity Pathway Analysis was used to analyze cellular pathways with differentially expressed genes. The *P* values for DEGs (Fold changes > 2 and FDR < 0.05) were then uploaded to Ingenuity Pathway Analysis (Qiagen), which was used to analyze the cellular pathways determined by the differentially expressed genes. No exclusion of genes was made for the cellular pathway analyses.

### Immunohistochemistry

Animals were deeply anesthetized using CO_2_ and transcardially perfused with PBS, followed by 4% paraformaldehyde (PFA) in PBS. Brains were post-fixed in 4% PFA overnight at 4 °C, and then cryoprotected using 30% sucrose in PBS for at least 24 h, followed by freezing and embedding in Tissue Tek OCT medium (Sakura Finetek USA Inc., CA, United States). A cryostat was used to collect 40-μm-thick coronal sections. All staining between groups used the same master solution mix of blocking buffer and antibodies. Immunohistochemistry was performed side by side between groups. Free-floating sections were washed in PBS and subsequently incubated in blocking buffer (0.5% Triton X-100, 5% normal goat serum, in PBS) for ∼2 h at room temperature. Sections were then incubated overnight (∼16 h) at 4°C in the primary antibodies diluted in blocking buffer. The primary antibodies were as follows: anti-eGFP (chicken polyclonal, GFP-1020, Aves, 1:1,000), anti-Cre recombinase (mouse monoclonal, MAB3120, Millipore, 1:200), anti-parvalbumin (mouse monoclonal, PV235, Swant, 1:1,000 or guinea pig polyclonal, GP72, Swant, 1:1,000) and anti-mCherry (mouse monoclonal, 3A11, DSHB (UIOWA), 1:500). After incubation, sections were washed three times in PBS and incubated with Alexa-fluor-conjugated secondary antibodies (goat anti-guinea pig, A-11073, Invitrogen, 1:5,000; goat anti-rabbit (31460) and goat anti-mouse (31430), Thermo Fisher Scientific, 1:5,000). After secondary incubation, sections were washed in PBS three times and mounted on glass slides with hard set Vectashield (Vector Labs, CA) for microscopy. Confocal images were obtained on a Zeiss LSM 710 confocal imaging system (Carl Zeiss Microscopy, Thornwood, NY) using a 20 × /0.8 N.A. air or a 100 × /1.4 N.A. oil-immersion objectives (Carl Zeiss Microscopy, Thornwood, NY). Gain, exposure time, and all other related settings were constant throughout each experiment. All image groups were processed in parallel using Fiji.

### Western Blot

Mouse hippocampal tissues were lysed with a lysis buffer (Pierce IP Lysis Buffer, 87788, Thermo Fisher Scientific) supplemented with a protease and phosphatase inhibitor cocktail (78442, Thermo Fisher Scientific). The tissue lysates were homogenized with a Tissue Grinder (10 strokes, 02-911-529, Thermo Fisher Scientific) and centrifuged at 800 × *g* for 5 min. Protein levels in the supernatant were measured by the BCA method. The samples were mixed with the standard protein sample buffer and boiled on a hot plate for 2 min, and subjected to SDS-PAGE with 4–20% Novex Tris-Glycine gels (Sigma-Aldrich), followed by protein transfer onto a nitrocellulose membrane. The membranes were Immunoblotted with incubation of anti-Ahnak (rabbit polyclonal, RU2024, 1:5,000) or Anti-Gapdh (mouse monoclonal, MAB374, Millipore, 1:5,000) primary antibody followed by incubation of horseradish peroxidase-linked goat anti-rabbit or anti-mouse secondary antibody (1: 5,000, Thermo Fisher Scientific). Antibody binding was detected using the enhanced chemiluminescence immunoblotting detection system (Perkin Elmer LLC WESTERN LIGHTNING PLUS-ECL, Thermo Fisher Scientific) and Kodak autoradiography film. The bands were quantified by densitometry using NIH Image 1.63 software.

### RNAscope

One day following SI tests, animals were anesthetized and rapidly decapitated. Brains were quickly removed and fresh frozen in dry ice. Sections were cut at 20 μm, mounted on slides, and stored at −80°C. Sections were fixed in 4% PFA for 15 min, dehydrated in serial ethanol concentrations (50, 70, and 100%) and processed with the RNAscope Multiplex Fluorescent assay (320293, RNAScope, Advanced Cell Diagnostics, Inc., CA, United States). Sections were hybridized with a mixture of selective probes for parvalbumin (Mm-Pvalb, 421931) and Ahnak (Mm-Ahnak, 576971). Sections were then counterstained with DAPI and coverslipped. Confocal images were obtained on a Zeiss LSM 710 confocal imaging system using a 100 × /1.4 N.A. oil-immersion objective (Carl Zeiss Microscopy). Because each image contained only a few cells, multiple stack images were taken of each vDG and treated independently. Z-stacks were taken at 1 μm step size. Gain, exposure time, and all other related settings were constant for each quantified image. To generate a projection image for each PV cell, each set of stack projections was z-stacked with maximum intensity using Fiji. To minimize the Ahnak puncta from other cell types, regions-of-interest (ROIs) were made around only dense PV mRNA puncta to capture PV-expressing cytoplasmic area of PV neurons in the vDG. These ROIs were then used for puncta and cell count analysis using Fiji. Total *n* = 73–102 PV cells in vDG were analyzed per group. 2–3 sections per mouse and total 3–4 mice per group were analyzed.

### Statistics

All data are expressed as means ± SEM except violin plots and pie charts. Sample sizes for biochemistry, electrophysiology and CSDS were determined based on our empirical data accumulated in the laboratory and previous studies using CSDS (Berton et al., 2006; Krishnan et al., 2007; Menard et al., 2017). Although no methods were used to predetermine sample sizes for TRAP/RNA-seq experiment, our sample sizes are similar to those reported in previous publications of TRAP-RNA-seq experiments (Medrihan et al., 2017; Ayata et al., 2018; Azevedo et al., 2019, 2020; Badimon et al., 2020; Karayol et al., 2021; Stern et al., 2021). Sample sizes and statistical methods are provided in each figure legend. Statistical analysis was performed using the two-tailed unpaired Student’s *t*-test, one-way ANOVA, two-way ANOVA or repeated-measures two-way ANOVA (see Summary of statistical analysis in Supplementary Table 1). ANOVAs of paired Gaussian distributions were followed with Bonferroni’s *post hoc* test. The D′Agostino-Pearson omnibus normality test was used to determine normality. For non-Gaussian distributions of multiple groups, the Kruskal–Wallis test followed by Dunn’s multiple comparisons *post hoc* test was used. GraphPad Prism 6 or 8 was used for statistical analysis and graphical preparations. For *in vivo* electrophysiology, RNAscope, immnoblotting and behavioral tests, no cutoff of potential outliers was made for statistical analysis. For TRAP/RNA-seq dataset, cutoffs for DEGs in TRAP/RNA-seq analysis were fold Change > 2 and FDR < 0.05.*p* < 0.05 was considered the cutoff for statistical significance. Statistical significance is shown as *p < 0.05, ^**^p < 0.01, ^***^p < 0.001, ^****^p < 0.0001, otherwise indicated in figures or figure legends.

## Results

### PV Neuron Activity in vDG Is Associated With Behavioral Responses to CSDS

**FIGURE 1:**
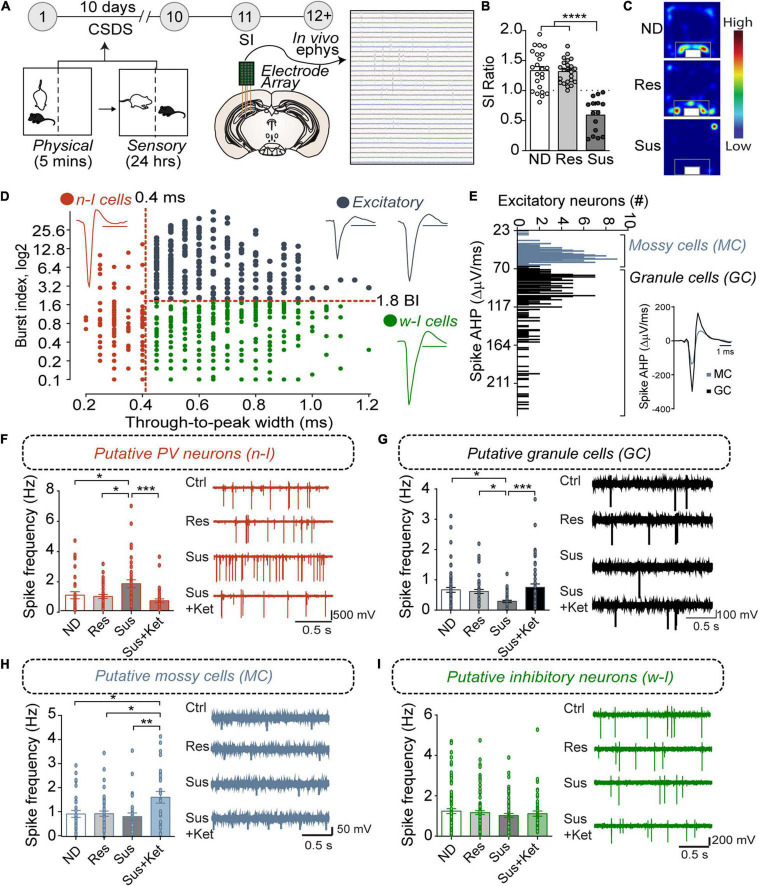
PV*^vDG^* neuron activity is altered by CSDS and associated with behavioral outcome. **(A)** Diagram and timeline of CSDS and intra-vDG electrophysiological recordings. **(B)** Representative animals depicting separation of animals into non-defeated control (ND), resilient (Res, SI ratio ≥ 1), and susceptible (Sus, SI ratio < 1) groups [one-way ANOVA, Non-defeated (*n* = 23), Res (*n* = 23) and Sus (*n* = 15)]. **(C)** Example heatmaps displaying time spent in the SI arena with caged aggressor. **(D)** All neurons detected (*n* = 772) went through a first stage classification based on the through-to-peak latency and burst index (BI) to distinguish putative excitatory cells (black dots, *n* = 336, BI > 1.8), narrow-waveform putative PV neurons (red dots, *n* = 142, latency ≤ 0.4 ms) and wide-waveform putative interneurons (green dots, *n* = 294, latency > 0.4 ms). Scale bars for the representative waveforms represent 1 ms. **(E)** Excitatory neurons were further classified in putative mossy cells (*n* = 133, AHP < 70) and putative granule cells (*n* = 203, AHP > 70) based on the bimodal distribution of the AHP current, measured from the first derivative of the spike. **(F–I)** Histograms show frequency (mean ± SEM) of the putative neuronal types analyzed in the respective experimental groups. Each black dot represents a neuron. **(F)** Susceptibility is associated with an increase in PV neuron spike frequency that is reversed by Ketamine (*n* = 31/7 (neurons/mice) ND mice; 36/7 resilient mice, 37/5 susceptible mice, and 38/3 for susceptible mice treated with ketamine). **(G)** Susceptibility is associated with decreases in GC spike frequency that is reversed by Ketamine [*n* = 61/7 (neurons/mice) ND mice, 47/7 resilient mice, 41/5 susceptible mice, and 54/3 susceptible mice treated with ketamine]. **(H)** Ketamine increases MC spike frequency [*n* = 31/7 (neurons/mice) ND mice, 49/7 for the resilient mice, 27/5 for the susceptible mice, and 26/3 susceptible mice treated with ketamine. **(I)** CSDS or Ketamine did not alter spike frequency in other inhibitory neurons (*n* = 85/7 (neurons/mice) ND mice, 97/7 resilient mice, 62/5 susceptible mice and 50/3 susceptible mice treated with ketamine). *p < 0.05, **p < 0.01, ***p < 0.001, one-way ANOVA with Bonferroni’s *post hoc* comparison.

The vDG is involved in mood control, stress responses, and antidepressant actions (Kheirbek et al., 2013; Anacker et al., 2018; Shuto et al., 2020). To assess the role of vDG neurons in stress-induced behavioral outcomes, we recorded vDG neuronal activity in mice that underwent CSDS (Figure 1A). The CSDS paradigm uses ethologically relevant stressors including physical defeat by and sensory contact with aggressor mice for 10 days to evoke social avoidance and anhedonic-like behavior, as measured by social interaction (SI) with a novel aggressor mouse and sucrose consumption (Krishnan et al., 2007; Golden et al., 2011). The SI test produces an SI ratio of the time spent in the interaction zone in the presence or the absence of a novel aggressor to identify susceptible (SI ratio below 1) and resilient (SI ratio equal to and greater than 1) groups (Figures 1B,C and Supplementary Figure 1). Non-defeated control mice remained housed in pairs without social defeat or contact with aggressors and display SI ratios similar to resilient mice.

**FIGURE 2:**
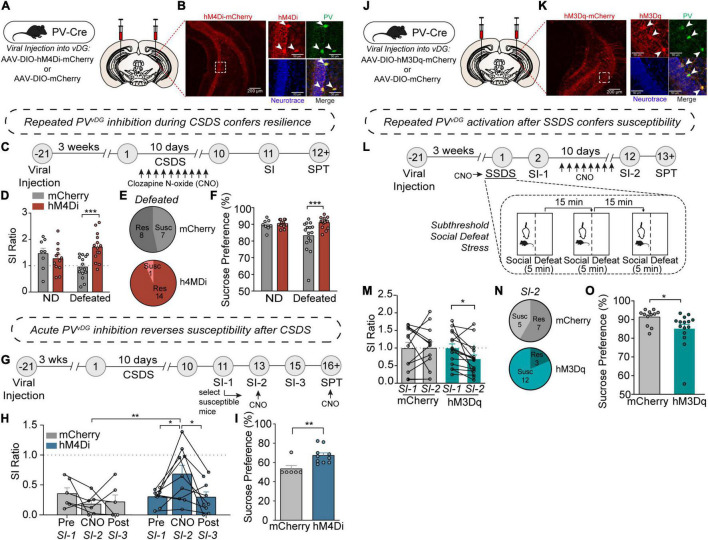
PV neuron activity in the vDG mediates resilience or susceptibility to CSDS. **(A)** Viral injection strategy for expression of hM4Di-mCherry or mCherry in vDG of PV-Cre mice. **(B)** Representative images showing hM4Di-mCherry expression in the vDG (left image; scale bar, 200 μm) and high magnification images of a dotted rectangular region showing selective expression of hM4Di-mCherry in PV neurons (right images; scale bar, 50 μm). **(C)** Timeline of repeated CNO treatments during CSDS and behavioral experiments. **(D)** Repeated CNO-treatment resulted in increased SI ratio [two-way ANOVA, Non-defeat (ND): mCherry (*n* = 8) and hM4Di (*n* = 12), Defeated: mCherry (*n* = 15) and hM4Di (*n* = 14)]. **(E)** Pie charts showing the number of resilient and susceptible animals after repeated injections of CNO during CSDS. **(F)** Chronic-CNO treatment during CSDS resulted in hM4Di mice displaying increased sucrose compared to mCherry controls [two-way ANOVA, Non-defeat: mCherry (*n* = 8) and hM4Di (*n* = 12), Defeated: mCherry (*n* = 16) and hM4Di (*n* = 16)]. **(G)** Timeline of CSDS, acute CNO treatments and behavioral experiments. **(H)** Acute CNO-treatment resulted in increased SI ratio only in hM4Di but not in mCherry control group during SI-2 test [two-way repeated measures ANOVA, mCherry (*n* = 6) and hM4Di (*n* = 9)]. **(I)** After acute-CNO treatment, hM4Di group displayed increased sucrose preference in SPT compared to mCherry control group [unpaired two-tailed *t*-test, mCherry (*n* = 6) and hM4Di (*n* = 10)]. **(J)** Viral injection strategy for expression of hM3Dq-mCherry or mCherry in vDG of PV-Cre mice. **(K)** Representative images showing hM3Dq-mCherry expression in the vDG (left image; scale bar, 200 μm) and high magnification images of a dotted rectangular region showing selective expression of hM3Dq-mCherry in PV neurons (right images; scale bar, 50 μm). **(L)** Timeline of SSDS, CNO treatments and behavioral experiments, and diagram of SSDS. **(M)** Comparison of SI ratio obtained from SI-1 (tested after an acute injection of CNO immediately followed by SSDS) versus SI-2 (tested after repeated daily injections of CNO for 10 days). After chronic injections of CNO, hM3Dq mice displayed increased social avoidance compared to mCherry controls [two-way repeated measures ANOVA, mCherry (*n* = 12) and hM3Dq (*n* = 15)]. **(N)** Pie charts showing the number of resilient and susceptible animals resulting from repeated injections of CNO after SSDS. **(O)** After repeated injections of CNO, hM3Dq mice displayed decreased sucrose preference compared to mCherry controls in SPT [unpaired two-tailed *t*-test, mCherry (*n* = 12) and hM3Dq (*n* = 15)]. Data are expressed as mean ± SEM, and individual data points are depicted. *Post hoc* Bonferroni’s multiple comparisons were used for ANOVA. *p < 0.05, **p < 0.01, ***p < 0.001.

To examine whether CSDS elicited adaptations in basal neuronal activity within the vDG, we recorded vDG neurons using *in vivo* high-density silicon probe recordings in anesthetized animals. Harnessing known characteristics of firing dynamics, we classified and separated discrete neuron classes. For example, neurons were classified on the basis of the width (through-to-peak latency) of the waveform of their spikes (Figure 1D, see section “Materials and Methods”) into “narrow” and “wide” waveform neurons to separate putative fast-spiking PV-positive neurons from other cell classes in the hippocampus (Stark et al., 2013). Additionally, the analysis of bursting, a marker of internal firing dynamics that distinguishes wide waveform putative excitatory from inhibitory neurons in the vDG, further separated the neurons into wide-inhibitory neurons and excitatory neurons (Senzai and Buzsaki, 2017). Furthermore, mossy cells have a small AHP current following an action potential (Scharfman and Myers, 2012) compared to granule cells (see section “Materials and Methods”) and, based on the bimodal distribution of the amplitude of the AHP current, we further separated the excitatory neurons in putative mossy cells and putative granule cells (Figure 1E).

Our results showed that, in susceptible mice, the firing frequency of putative PV neurons was significantly increased in susceptible mice compared to non-defeated or resilient mice (Figure 1F). In contrast, the firing frequency of putative granule cells was significantly decreased in susceptible mice compared to the frequency in non-defeated or resilient mice (Figure 1G). No difference, however, was found in the firing frequency of mossy cells between non-defeated, susceptible, or resilient groups (Figure 1H). To examine whether CSDS-induced adaptations in neuron activity could be reversed by known fast-acting antidepressants (Kim et al., 2019), we administered ketamine in susceptible mice and recorded neuronal activity. Ketamine ameliorated the increase in firing frequency of putative PV neurons and restored them to comparable levels to non-defeated or resilient mice (Figure 1F). In addition, ketamine treatment increased firing rates in putative granule and mossy cells (Figures 1G,H). However, the firing frequency of other inhibitory neurons (CCK, SOM etc.) remained unchanged in resilient and susceptible mice with or without ketamine treatment as compared to control mice (Figure 1I). Together, these data suggest that CSDS-induced neuron activity changes of PV*^vDG^* and granule cells (GC*^vDG^*) may play a critical role in divergent behavioral responses to stress. The opposing directionality in CSDS-induced changes in PV neurons and GCs likely arise from the perisomatic inhibition onto large GC populations by PV neurons (Kraushaar and Jonas, 2000; Struber et al., 2015). Our previous studies suggested that inhibition of calcium signaling or neuronal activity selectively in PV neurons induces an antidepressant-like behavioral phenotype (Lee et al., 2015; Jin et al., 2020), and this notion is also supported by the reduction of PV*^vDG^* neuron activity by ketamine (Figure 1F). We thus sought to determine whether inhibition or activation of PV*^vDG^* was capable of conferring or suppressing resilience to CSDS, respectively.

### PV Activity in vDG Mediates Behavioral Responsivity to CSDS

To evaluate whether PV*^vDG^* neuronal activity mediates behavioral responses to CSDS, we employed the use of DREADDs (designer receptor exclusively activated by designer drugs) (Roth, 2016) to manipulate PV*^vDG^* neuronal activity *in vivo*. The vDG of PV-Cre mice were bilaterally injected with Cre-dependent hM4Di-mCherry or empty vector mCherry (Figure 2A) to selectively express hM4Di-mCherry in PV*^vDG^* (Figure 2B). First, we assessed whether repeated chemogenetic inhibition of PV neurons concurrent with CSDS would affect behavioral divergence. Mice expressing hM4Di or mCherry in PV*^vDG^* underwent CSDS, during which the DREADD agonist clozapine *N*-oxide (CNO) was administered 30 min prior to each defeat session to suppress neuronal activity during and after each social stress encounter (Figure 2C). Interestingly, repeated inhibition of PV*^vDG^* neuronal activity promoted resilience evident from an increased SI ratio (Figures 2D,E and Supplementary Figure 2A). Additionally, repeated CNO-treatment resulted in mitigated stress-induced anhedonic-like behavior in the sucrose preference test (SPT) (Krishnan et al., 2007) in mice expressing hM4Di in PV*^vDG^* compared to mCherry (Figure 2F).

Given that stress-susceptible mice administered with ketamine displayed PV*^vDG^* firing rates similar to non-defeated and stress-resilient mice (Figure 1F), we questioned whether acute inhibition of PV*^vDG^* might be sufficient to reverse social avoidance and anhedonic-like behavior in stress-susceptible mice. To address this, mice were exposed to CSDS to generate susceptible mice expressing either hM4Di or empty vector mCherry as a control. 48 h after the initial SI test (SI-1), only susceptible mice (SI ratio < 1.0) were administered with CNO 30 min before a subsequent SI-test (SI-2) (Figure 2G). Remarkably, acute CNO administration was capable of attenuating susceptibility as evident by an increased SI ratio (Figure 2H) and time spent in the interaction zone with the aggressor (Supplementary Figure 2B). This amelioration of social avoidance returned to SI scores similar to those in SI-1 when tested another 48-h later (SI-3) before which CNO was not administered (Figure 2H), suggesting that CNO-mediated PV inhibition acutely and transiently reverses behavioral susceptibility. To test whether acute inhibition of PV*^vDG^* can attenuate stress-induced anhedonic-like behavior, we administered CNO during SPT. Indeed, acute administration of CNO increased sucrose preference in mice expressing hM4Di compared to mCherry control mice (Figure 2I).

We next evaluated whether chemogenetic activation of PV*^vDG^* would be sufficient to produce stress susceptibility. We bilaterally injected Cre-dependent AAV expressing hM3Dq-mCherry or empty vector mCherry into the vDG of PV-Cre mice (Figure 2J) to selectively express hM3Dq-mCherry in PV*^vDG^* (Figure 2K). We adopted a subthreshold social defeat stress (SSDS) paradigm that uses 3 social stress sessions in 1 day (Cheng et al., 2019) and tested the effect of acute activation of PV*^vDG^* by CNO during SSDS or repeated activation after SSDS on behavior (Figure 2L). While acute CNO treatment during SSDS did not cause any significant difference between hM3Dq mice and mCherry control mice (Supplementary Figures 2C,D), subsequent repeated daily activation of PV*^vDG^* neurons after SSDS resulted in behavioral susceptibility indicated by a decreased SI ratio (Figures 2M,N and Supplementary Figure 2E) and reduced sucrose preference (Figure 2O). Repeated CNO administration in hM3Dq mice naïve to previous stressors, however, failed to elicit this decrease (Supplementary Figures 2F–H). These data suggest that while repeated or acute inhibition is sufficient to drive resilience to social stress, activation of these neurons must occur repeatedly over the course of many days to drive stress-susceptibility, potentially indicative of what occurs during CSDS.

### Molecular Adaptations in Hippocampal PV Neurons Are Associated With Divergent Behavioral Consequences After CSDS

**FIGURE 3:**
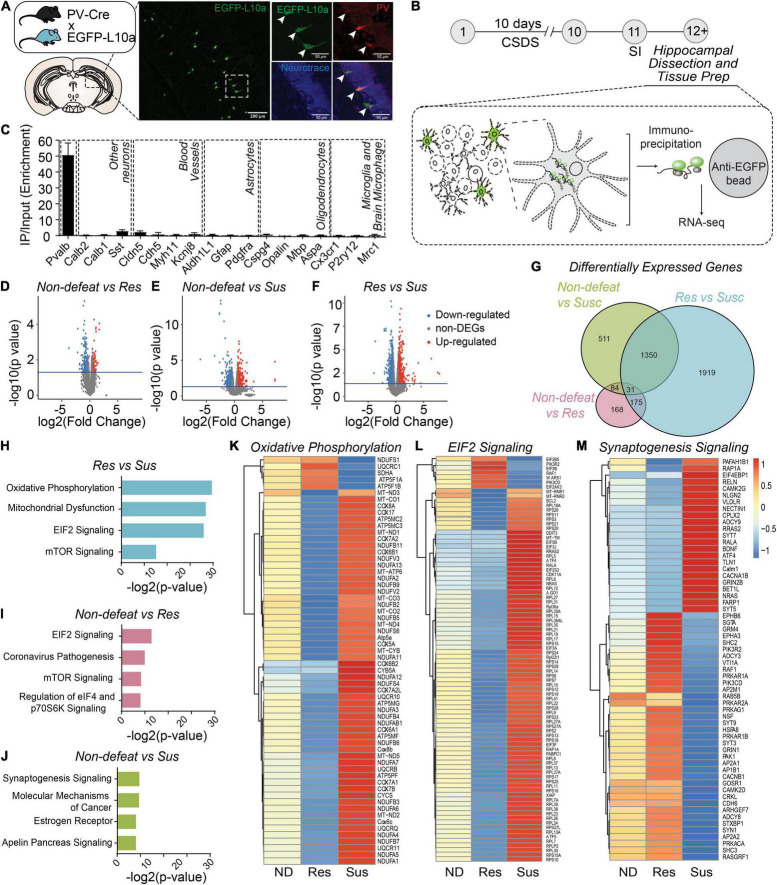
Molecular adaptations in hippocampal PV neurons are associated with divergent behavioral consequences after CSDS. **(A)** Diagram of transgenic PV-specific EGFP-L10a line for TRAP. Representative images showing EGFP-L10a expression in the vDG (left image; scale bar, 200 μm) and high magnification images of a dotted rectangular region showing selective expression of EGFP-L10a in PV neurons (right images; scale bar, 50 μm). **(B)** Timeline of CSDS and subsequent hippocampal dissection, TRAP and RNAseq. **(C)** Enrichment of marker genes in TRAP/RNAseq data. **(D–F)** Volcano plots of DEGs in Non-defeated (ND) vs Res **(D)**, ND vs Sus **(E)** and Res vs Sus **(F)** comparisons. **(G)** Venn diagram for DEGs (cutoff, *p* < 0.05). **(H–J)** Top 4 pathways significantly altered in Res vs Sus **(H)**, ND vs Res **(I)**, ND vs Sus **(J)** comparisons. **(K–M)** Heat maps of DEGs in the top pathway in each comparison. Mean value of gene expression was used [(ND (*n* = 4), Res (*n* = 4) and Sus (*n* = 3)].

To determine whether molecular adaptations in hippocampal PV neurons were associated with resilience or susceptibility to CSDS, we generated PV neuron-specific expression profiles using the TRAP/RNA-seq approach (Heiman et al., 2008). We used a Cre recombinase-dependent TRAP line crossed with PV-Cre line to express the ribosomal protein L10a fused to enhanced green fluorescent protein (EGFP) selectively in PV neurons (Figure 3A). After CSDS, mouse hippocampal homogenates of non-defeated, resilient, and susceptible mice were used for immunoprecipitation of EGFP-tagged polysomes from PV neurons, and polysome-attached mRNAs were isolated and sequenced (Figure 3B). Importantly, PV-neuron specific TRAP revealed high selectivity, evident by high enrichment of PV mRNA, but not markers of other neuronal types, neuroglia and cell types in blood vessels, suggesting tight selectivity (Figure 3C).

RNA-seq revealed 458 differentially expressed genes (DEGs) between non-defeated and resilient mice, 1976 DEGs in non-defeated and susceptible mice, and 3475 DEGs in resilient and susceptible mice (Figures 3D–G). Pathway-analysis with DEGs was performed (Figures 3H–J), and between resilient and susceptible mice or non-defeated, genes associated with oxidative phosphorylation (Figure 3K), mitochondrial dysfunction (Supplementary Figure 3B), EIF2 signaling (Figure 3L) or mTOR signaling (Supplementary Figure 3B) were differentially expressed. In contrast, changes in expression of genes associated with synaptogenesis were most divergent between non-defeated and susceptible mice (Figures 3J,M). These results suggest that alterations of mitochondrial, protein synthesis, cell metabolism and synaptic pathways in hippocampal PV neurons may drive adaptive changes in PV*^vDG^* neuronal activity and behavioral responses to CSDS.

### Chronic Stress Alters Expression of Ahnak in the Hippocampus

**FIGURE 4:**
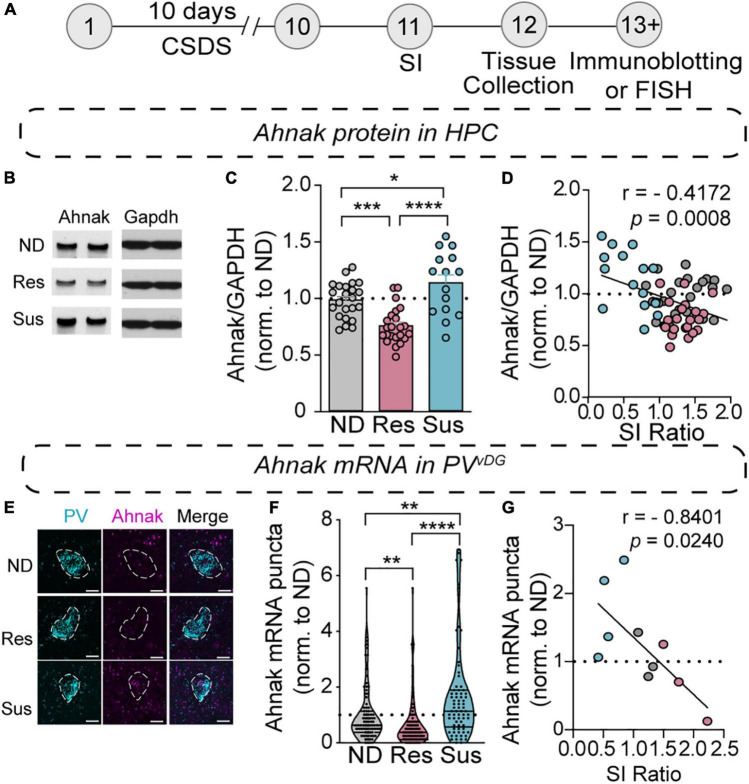
Chronic social stress alters Ahnak expression in the hippocampus. **(A)** Diagram and timeline of CSDS and tissue collection. **(B–D)** Immunoblotting of Ahnak protein using hippocampal lysates. **(B)** Representative images of immunoblotting of Ahnak and Gapdh as a control. **(C)** Quantification of immunoblots [one-way ANOVA, Non-defeated (*n* = 23 mice), Res (*n* = 23 mice) and Sus (*n* = 15 mice)]. **(D)** Hippocampal Ahnak is inversely correlated with SI ratio (pearson *r*: *r* = –0.4172, *p* = 0.0008, *n* = 61 mice) **(E)** Representative images PV and Ahnak mRNA expression using RNAScope. Scale bar, 5 μm. **(F)** Ahnak mRNA expression in vDG PV neurons from Non-defeat, Res and Sus groups (Kruskal–Wallis test with *post hoc* Dunn’s multiple comparisons: Non-defeated (*n* = 102 cells), Res (*n* = 94 cells) and Sus (*n* = 73 cells). **(G)** Ahnak mRNA expression in vDG PV neurons is inversely correlated with SI ratio (pearson *r*: *r* = –0.8401, *p* = 0.0240, *n* = 10 mice). All data were normalized to ND group. *p < 0.05, **p < 0.01, ***p < 0.001, ****p < 0.0001.

Our previous study identified Ahnak as a regulator of “depressive”-like behavior, potentially in part due to its role in trafficking L-type VGCCs to the surface, positioning Ahnak to regulate neuronal activity responses (Jin et al., 2020). Remarkably, we find that susceptible mice have increased Ahnak mRNA in our TRAP/RNAseq dataset (*p* = 0.0399, Supplementary Table 2). To confirm whether behavioral responses to CSDS are associated with changes in Ahnak expression, we subjected mice to CSDS followed by SI-test and brain tissue collection (Figure 4A). First, we performed immunoblotting for Ahnak protein in the whole hippocampus. Ahnak protein levels were reduced in the hippocampus of resilient mice but increased in the hippocampus of susceptible mice, compared to non-defeated control mice (Figures 4B,C). Hippocampal Ahnak levels were also inversely correlated with SI ratio (Figure 4D) and time spent in the interaction zone containing an aggressor mouse (Supplementary Figure 4A). These data indicate that CSDS induces alterations of hippocampal expression of Ahnak in opposing directions, depending on the behavioral response of individual mice.

**FIGURE 5:**
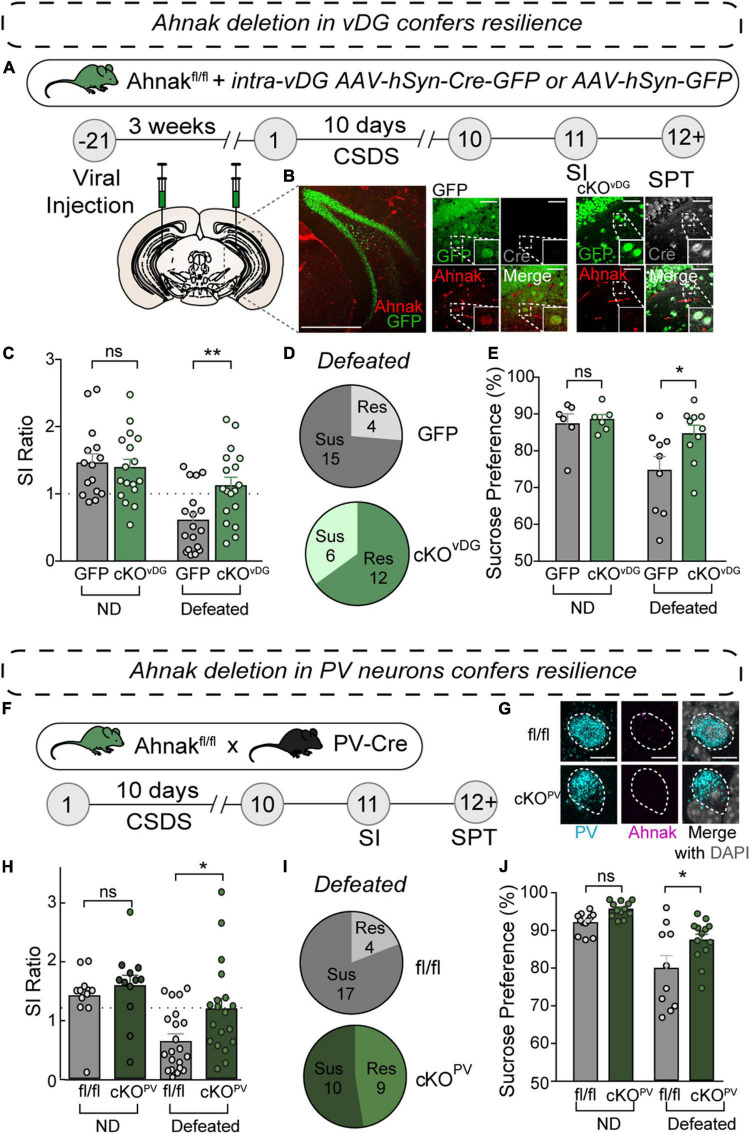
Ahnak deletion in vDG or PV neurons confers resilience to CSDS. **(A)** Diagram and timeline for conditional knockout approach using stereotaxic AAV injections of AAV-Cre-GFP or AAV-GFP control. **(B)** Representative confocal image of coronal brain section stained for GFP and Ahnak, depicting deletion of Ahnak selectively in vDG neurons. Scale Bar, 500 μm (left), 50 μm (right). **(C)** Ahnak cKO*^vDG^* mice display a higher SI ratio than GFP-controls (Cont) [two-way ANOVA, Non-defeat (ND): Cont (*n* = 16) and cKO*^vDG^* (*n* = 17), Defeated: Cont (*n* = 19) and cKO*^vDG^* (*n* = 18)]. **(D)** Pie charts showing the number of resilient and susceptible animals resulting after CSDS. **(E)** Ahnak cKO*^vDG^* mice display decreased stress-induced anhedonic-like behavior in the sucrose preference test after CSDS [two-way ANOVA, ND: Cont (*n* = 6) and cKO*^vDG^* (*n* = 6), Defeated: Cont (*n* = 9) and cKO*^vDG^* (*n* = 10)]. **(F)** Behavioral timeline and diagram of conditional knockout approach for Ahnak cKO*^PV^*. **(G)** Confocal images showing absence of Ahnak mRNA in PV*^vDG^* neurons in mice with a conditional deletion in PV neurons (cKO*^PV^*). **(H)** After CSDS, cKO*^PV^* mice display a higher SI ratio compared to control mice (fl/fl) [two-way ANOVA, ND: fl/fl (*n* = 12) and cKO*^PV^* (*n* = 12), Defeated: fl/fl (*n* = 21) and cKO*^PV^* (*n* = 19)]. **(I)** Pie charts showing the number of resilient and susceptible animals resulting after CSDS. **(J)** Ahnak cKO*^PV^* mice display increased sucrose preference after CSDS compared to controls [two-way ANOVA, ND: fl/fl (*n* = 12) and cKO*^PV^* (*n* = 12), Defeated: fl/fl (*n* = 10) and cKO*^PV^* (*n* = 13)]. Data are expressed as mean ± SEM and individual data points are depicted. *Post hoc* Bonferroni’s multiple comparisons were used for ANOVA. *p < 0.05, **p < 0.01 and ns, non-significant.

Our previous study showed that PV neuron-selective Ahnak KO mice displayed antidepressant-like behavior (Jin et al., 2020). However, homogenates used for immunoblotting include protein of all cell types and subregions within the hippocampus. To evaluate whether CSDS induces alterations of Ahnak expression within PV neurons, we turned to evaluating Ahnak mRNA within PV*^vDG^* in non-defeated, stress-resilient and -susceptible mouse brains using RNAscope, a fluorescent RNA *in situ* hybridization assay (Wang et al., 2012). We used a selective probe for Ahnak mRNA together with a selective probe for PV mRNA in order to indicate the location of PV neurons and quantify Ahnak expression in PV*^vDG^* (Figure 4E). The number of puncta of Ahnak per PV*^vDG^* was significantly increased in susceptible mice as compared to the number in non-defeated controls or resilient mice, while the resilient mice displayed a significant decrease in Ahnak expression compared to non-defeated control mice (Figure 4F). Additionally, Ahnak mRNA in PV*^vDG^* is inversely correlated with SI Ratio and interaction zone containing an aggressor mouse (Figure 4G and Supplementary Figure 4B). This result is consistent with a bidirectional change of Ahnak protein in the hippocampal tissues of susceptible versus resilient mice compared to non-defeated control mice. These results altogether indicate that hippocampal Ahnak expression in PV*^vDG^* neurons is altered by CSDS, particularly upregulated in stress-susceptible mice.

### Ahnak Deletion in vDG or PV Neurons Confers Behavioral Resilience to CSDS

To investigate whether Ahnak is required for CSDS-induced behavioral susceptibility, our approach was twofold: to assess the behavioral consequence of Ahnak deletion 1) in the vDG and 2) selectively in PV neurons. To delete Ahnak in a region-selective manner, we generated Cre-loxP-mediated ventral dentate gyrus (vDG)-specific Ahnak knockout (KO) mice. We used floxed Ahnak mice (Jin et al., 2020) and injected adeno-associated virus (AAV) expressing Cre recombinase fused to GFP, or empty vector GFP as a control, under a human synapsin promoter into the ventral dentate gyrus (vDG) to selectively delete Ahnak in vDG neurons (cKO*^vDG^*) (Figure 5A). Immunohistochemical staining of GFP, Cre recombinase and Ahnak reveal selective KO of Ahnak in neurons infected by AAV-Cre-GFP compared to AAV-GFP (Figure 5B). In response to CSDS, Ahnak cKO*^vDG^* resulted in increases of SI ratio (Figure 5C) and time interacting with an aggressor compared to control mice (Supplementary Figure 5A), generating a greater portion of resilient mice (Figure 5D). Additionally, defeated Ahnak cKO*^vDG^* mice display mitigated anhedonic-like behavior as measured by increased sucrose consumption compared to defeated control mice (Figure 5E). However, non-defeated Ahnak cKO*^vDG^* mice and control mice display comparable behaviors in SI test (Figure 5C and Supplementary Figure 5A) and SPT (Figure 5E).

We next investigated whether Ahnak deletion selectively in PV neurons affects stress-induced behavioral responses. To delete Ahnak selectively in PV neurons, we generated offspring from crossing floxed Ahnak mice with a PV neuron-specific Cre recombinase line (cKO*^PV^*) (Figure 5F), which results in PV-selective Ahnak KO (Jin et al., 2020; Figure 5G). We exposed Ahnak cKO*^PV^* mice (f/f; Cre-positive) and control mice (f/f; Cre-negative) to CSDS and performed SI test and SPT (Figure 5F). Defeated Ahnak cKO*^PV^* mice showed an increased SI ratio compared to the defeated control fl/fl mice (Figure 5H and Supplementary Figure 5B), yielding a greater portion of resilient mice (Figure 5I). Furthermore, defeated Ahnak cKO*^PV^* mice display increased sucrose consumption compared to the defeated control group suggesting an attenuation of the CSDS-induced anhedonic-like behavior (Figure 5J). Non-defeated Ahnak cKO*^PV^* mice and control mice display comparable behaviors in SI test (Figure 5H and Supplementary Figure 5B) and SPT (Figure 5J). Astoundingly, these results are consistent with those found with conditional deletion of Ahnak in the vDG (cKO*^vDG^*). Altogether, these data suggest that the increases in Ahnak expression observed in PV*^vDG^* of susceptible mice (Figures 4E–G) is required for generating behavioral susceptibility from CSDS.

Since Ahnak and increased PV activity are both required for behavioral susceptibility, we next aimed to assess whether Ahnak deletion in PV neurons affects the activity of PV neurons, we performed whole-cell current-clamp recordings of PV*^vDG^* using acute hippocampal slices from Ahnak cKO*^PV^* and control mice (Supplementary Figure 6A). The firing frequency induced by current injection in PV neurons in Ahnak cKO*^PV^* mice was significantly reduced compared to the control group (Supplementary Figures 6B,C), suggesting a role of Ahnak in the modulation of PV firing. Analyses of action potential properties indicate that Ahnak cKO*^PV^* does not influence the voltage threshold, action potential amplitude and afterhyperpolarization (AHP), but it increases the half-amplitude width (Supplementary Figures 6D–H). These results implicate that the decrease of excitability of PV neurons by Ahnak deletion may contribute to behavioral resilience or impede susceptibility after CSDS.

## Discussion

Here, we demonstrate that PV*^vDG^* neuronal activity and alterations in hippocampal Ahnak expression are critical for generating susceptibility or resilience to CSDS. Our study complements previous studies using rodent chronic stress paradigms to identify various cellular and molecular adaptations including ion channels, synaptic proteins, transcription factors, microRNAs and epigenetic regulators in key neural circuits regulating stress susceptibility or resilience including amygdala, thalamic, and hippocampal circuits as well as catecholaminergic circuits (Russo et al., 2012; Han and Nestler, 2017; Cathomas et al., 2019). In this study, we identified a causal relationship of neuronal activity of PV*^vDG^* in behavioral susceptibility or resilience to CSDS. We also identified that many divergent gene expression patterns emerged between resilient mice and susceptible mice as compared to non-defeated mice. For instance, DEGs associated with oxidative phosphorylation and EIF2 signaling are largely decreased in resilient mice but increased in susceptible mice compared to non-defeated mice (Figures 3K,L). However, DEGs associated with synaptogenesis or mTOR signaling comprised of a mixture of upregulated or downregulated genes across both resilient and susceptible mice (Figure 3M and Supplementary Figure 3B). These results suggest that active molecular changes involved in mitochondrial function, energy metabolism, protein translation and synaptic plasticity underlie adaptations of PV neuronal firing in stress-susceptible and resilient groups. We also identified divergent alterations of Ahnak in hippocampal lysates and PV*^vDG^* (Figure 4). However, the pattern of firing changes of PV neurons is somewhat different from that of gene changes. PV neuronal firing is increased only in susceptible mice, but the firing rate in resilient mice is comparable to that of non-defeated controls (Figure 1), reminiscent of previous observations in ventral tegmental area (VTA) neurons (Cao et al., 2010; Chaudhury et al., 2013; Friedman et al., 2014). Only stress-susceptible mice display increased firing of dopaminergic neurons projecting from the VTA to the nucleus accumbens (NAc) and decreased firing of dopaminergic neurons projecting from the VTA to the mPFC (Cao et al., 2010; Chaudhury et al., 2013). Although significantly more genes are regulated in the VTA and NAc in resilient mice compared with susceptible mice (Krishnan et al., 2007), resilient mice display control-level firing activity in both of the aforementioned circuits (Friedman et al., 2014). This adaptation of firing activity in resilient mice has been explained by homeostatic adaptation of intrinsic properties through up-regulated hyperpolarization-activated current as an excitatory driving force in conjunction with upregulation of potassium channels as an inhibitory driving force for intrinsic excitability of neurons (Friedman et al., 2014). Thus, the DEGs observed in resilient mice in this study may evoke a homeostatic control mechanism to similarly maintain the firing of PV neurons at a comparable rate with non-defeated mice.

We used urethane-anesthetized mice in *in vivo* electrophysiology experiments (Figure 1). Previously, urethane was found to be a better anesthesia than ketamine for more successful sorting of neuronal spikes (Hildebrandt et al., 2017). However, urethane also moderately affects multiple neurotransmitter-gated ion channels including GABA channels (Hara and Harris, 2002), and it reduces the synaptic and spike activity of hippocampal neurons when compared to awake rodents (Yagishita et al., 2020). Thus, although a direct connection between urethane and stress was not previously shown, it is still possible that urethane anesthesia might influence spike patterns, being a limitation in our experiment. However, the relationship between PV neuronal firing and stress-susceptibility or resilience observed in *in vivo* recording is strongly supported by behavioral analyses in conjunction with chemogenetics (Figure 2) and gene expression changes involved in mitochondria oxidative phosphorylation (Figure 3).

Previously, we identified Ahnak as an endogenous regulator of L-type voltage-gated calcium channels (VGCCs) in mice (Jin et al., 2020). Human genetic studies implicate altered function of L-type VGCCs in the pathophysiology of multiple psychiatric disorders including major depressive disorder, bipolar disorder, schizophrenia and autism spectrum (Green et al., 2010; Liu et al., 2011; Bhat et al., 2012, Cross-Disorder Group of the Psychiatric Genomics Consortium[Corporate Author], 2013; Schizophrenia Working Group of the Psychiatric Genomics, 2014; Pinggera et al., 2015). L-type VGCCs have been implicated in the rapid antidepressant actions of ketamine (Lepack et al., 2016) and scopolamine (Yu et al., 2018). These observations raise a potential connection between Ahnak-mediated pathways and stress-induced depression-like behavior and its amelioration by antidepressants. In this study, we have observed that Ahnak level is reduced or elevated in the hippocampal PV neurons selectively in resilient or susceptible mice, respectively (Figure 4), and deletion of Ahnak in PV neurons facilitates behavioral resilience after CSDS (Figure 5). It is possible that these alterations in Ahnak contribute to, at least in part, the regulation of PV neuronal firing. This pivotal role of Ahnak in PV neurons for CSDS-induced behavior is presumably given by its regulation of L-type VGCCs. The N-terminal region of Ahnak binds to Ca_*v*_1.2, an L-type pore-forming α1 subunit, and its C-terminal region scaffolds the β subunit of VGCC and the p11/Anxa2 complex (Jin et al., 2020). Cell surface expression of Ca_*v*_1.2 and Ca_*v*_1.3 and L-type calcium current were significantly reduced in Ahnak KO neurons compared to wild-type controls (Jin et al., 2020). Because L-type VGCC-mediated calcium signaling mediates nuclear gene expression, synaptic plasticity and homeostatic control of neuronal circuitry (Simms and Zamponi, 2014), altered levels of Ahnak in PV neurons likely modulate calcium signaling-mediated neuronal adaptations. Notably, gene expression of most L-type VGCC subunits are not altered in comparison between stress groups with the exception of Cacnb1 gene (*p* = 0.0244 for Res vs Sus, Supplementary Table 2). Thus, we propose a model wherein changes of Ahnak expression alter trafficking of L-type VGCCs to the plasma membrane and thereby alter channel activity, possibly resulting in gene alterations relevant to mitochondria, protein synthesis and synaptogenesis pathways.

In the brain, Ahnak scaffolds p11, alterations of which is highly implicated in the pathophysiology and antidepressant actions (Svenningsson et al., 2013). Previously, we observed that proteins, but not mRNA levels, of p11 and Anxa2 were highly destabilized in the absence of Ahnak (Jin et al., 2020), suggesting that the p11-mediated pathways may also be involved in adaptations of PV neurons in response to CSDS. In this study, we have found that p11 mRNA is also significantly increased in susceptible mice compared to non-defeat control (mean RPKM 14.810 in Sus vs 11.144 in non-defeat, *p* = 0.0385) or resilient mice (mean RPKM 14.810 in Sus vs 10.126 in Res, *p* = 0.0058) in the TRAP dataset (Supplementary Table 2). P11 plays a role in gene expression by regulating SMACA3, a chromatin remodeling factor, and possibly together with Supt6, an RNA polymerase II binding partner and also known as a histone chaperone (Oh et al., 2013; Lu et al., 2020; Umschweif et al., 2021b). Thus, nuclear p11 pathways may contribute to the gene alterations associated with mitochondria, protein synthesis and synaptogenesis pathways that we identified in this study. In addition to the nuclear roles, recent studies indicate roles for p11 in intrinsic membrane excitability, which is mediated by gene regulation of Kv3.1β potassium channels in hippocampal PV neurons (Sagi et al., 2020) or HCN2 channels (hyperpolarization activated cyclic nucleotide gated potassium and sodium channel) in cholinergic interneurons in the nucleus accumbens (Cheng et al., 2019). Importantly, Kv3.1β inhibition has been suggested to play a role in the therapeutic actions of selective serotonin reuptake inhibitor (SSRI) antidepressants by suppressing PV*^vDG^* neurons *via* Gi-coupled 5-HT5A receptor-mediated signaling (Sagi et al., 2020). Notably, Gq-coupled mGluR5 receptor deletion selectively in PV neurons renders an antidepressant-like behavioral phenotype (Lee et al., 2015). Furthermore, pharmacological inhibition of mGluR5 and thereby disinhibition of PV neuron-mediated suppression of circuit activity underlies a fast-antidepressant-like activity of an mGluR5 antagonist (Lee et al., 2015). Consistent with these observations, we have also observed suppression of PV neuron firing in resilient mice as well as ketamine-treated susceptible mice (Figure 1F). Taken together, these studies suggest the PV inhibition as a common cellular mechanism associated with biological resilience as well as pharmacological actions of two different classes of antidepressants.

Interestingly, a recent study by Medrihan et al. (2020) suggested that decreased Kv3.1 channel and p11 function in PV*^vDG^* would confer susceptibility after stress (Medrihan et al., 2020). Although Ahnak cKO*^PV^* mice show resilience after CSDS (Figures 5F–J), p11 cKO*^PV^* mice show stress-susceptibility after SSDS (Medrihan et al., 2020), likely through many other downstream effectors of p11 in PV*^vDG^* including mGluR5, SMARCA3, Supt6 and 5-HT5A (Oh et al., 2013; Lee et al., 2015; Sagi et al., 2020). Additionally, the timing of modulation of PV*^vDG^* or vDG network activity prior, during, and after stress likely affect behavioral outcome. We show in this study that inhibition each day of chronic stress (Figures 2C–F), as well as acute inhibition after stress (Figures 2G–I), results in stress-resilience. However, Medrihan et al. observed that acute chemogenetic inhibition of vDG*^PV^* during SSDS increased stress-susceptibility in the social interaction test. Further studies monitoring the activity of PV*^vDG^* neurons during CSDS are necessary to fully understand their involvement in CSDS-induced behavioral responses. Importantly, most CSDS studies thus far primarily use male mice, limiting the implications arising from these studies. In recent years, however, CSDS models have been adapted for use in female mice (Takahashi et al., 2017; Harris et al., 2018; Newman et al., 2019; Yohn et al., 2019). Because of gender differences in the prevalence of mood disorders (Seney and Sibille, 2014; Riecher-Rossler, 2017), the use of these newer models and testing our findings in females is warranted.

It was recently discovered that increased adult hippocampal neurogenesis inhibits a population of stress responsive GC in vDG, conferring stress resilience (Anacker et al., 2018). Interestingly, Ahnak deletion promotes hippocampal neurogenesis (Shin et al., 2015). CSDS-induced hippocampal Ahnak reductions might promote hippocampal neurogenesis as an alternative mechanism for stress resilience. Notably, ∼25% reduction of Ahnak protein in hippocampal lysates in resilient mice (Figure 4C) cannot be explained solely by Ahnak reduction in PV interneurons, which constitutes at best ∼ 5% of total hippocampal neurons (Pelkey et al., 2017). Thus, our findings do not eliminate a potential involvement of Ahnak expressed in other neuronal types or non-neuronal cells in stress resilience. In fact, Ahnak is highly expressed in endothelial cells in blood vessels in the hippocampus (Gentil et al., 2005; Jin et al., 2020), and tight junctions therein provide the property of the blood-brain barrier (Daneman and Prat, 2015). Intriguingly, endothelial cells have been implicated as a target of CSDS, and CSDS-induced dysfunction of the BBB has been suggested as a mechanism underlying stress susceptibility (Menard et al., 2017). Thus, further understanding of the function of Ahnak in other cell types will advance our understanding of stress resilience. Altogether, our study establishes a foundation supporting Ahnak and PV interneurons as potential targets for manipulation of stress-related hippocampal physiology or neuropsychiatric disorders such as MDD.

## Data Availability Statement

RNA-seq data from Figure 3 have been deposited and are available from GEO (accession number: GSE184027). Additional data related to this article may be requested from the authors.

## Ethics Statement

The animal study was reviewed and approved by Rockefeller University IACUC committee.

## Author Contributions

DB and YK conceptualized and designed the entire study. LM performed and analyzed *in vivo* electrophysiological experiments. LM and KM performed and analyzed *ex vivo* electrophysiological experiments. DB and MC performed and analyzed all the behavioral experiments and RNAscope experiments. DB performed all stereotaxic surgeries. DB, MC, and JJ performed and analyzed all the immunoblotting experiments. MC, JL, and EA performed the tissue collection for TRAP. MC, JL, and WW performed the analysis for TRAP experiments. DB and YK wrote the manuscript and designed figures with collective input from all authors. YK supervised the entire study. All authors contributed to the article and approved the submitted version.

## Conflict of Interest

The authors declare that the research was conducted in the absence of any commercial or financial relationships that could be construed as a potential conflict of interest.

## Publisher’s Note

All claims expressed in this article are solely those of the authors and do not necessarily represent those of their affiliated organizations, or those of the publisher, the editors and the reviewers. Any product that may be evaluated in this article, or claim that may be made by its manufacturer, is not guaranteed or endorsed by the publisher.

## Footnotes

1
https://github.com/flatironinstitute/mountainlab
