# Supplementary material for: Molecular and Cellular Adaptations in Hippocampal Parvalbumin Neurons Mediate Behavioral Responses to Chronic Social Stress
Source: Front Mol Neurosci. 2022 Jun 24;15:898851. doi: 10.3389/fnmol.2022.898851 (PMC9268684; doi:10.3389/fnmol.2022.898851)
Supplement: Supplementary file 1 [file Data_Sheet_1.pdf]

Supplementary Figures

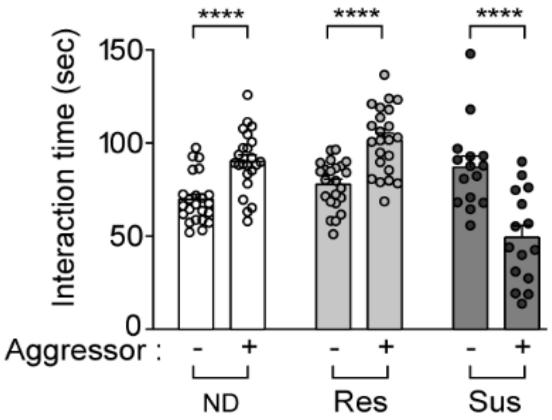

Supplementary Figure 1. **Interaction time in non-defeated control, resilient and susceptible groups after CSDS**. Time spent in the interaction zone without (-) and with (+) aggressor (two-way repeated measures ANOVA, Non-defeated (ND) (n=23), Resilient (Res) (n=23) and Susceptible (Sus) (n=15)).

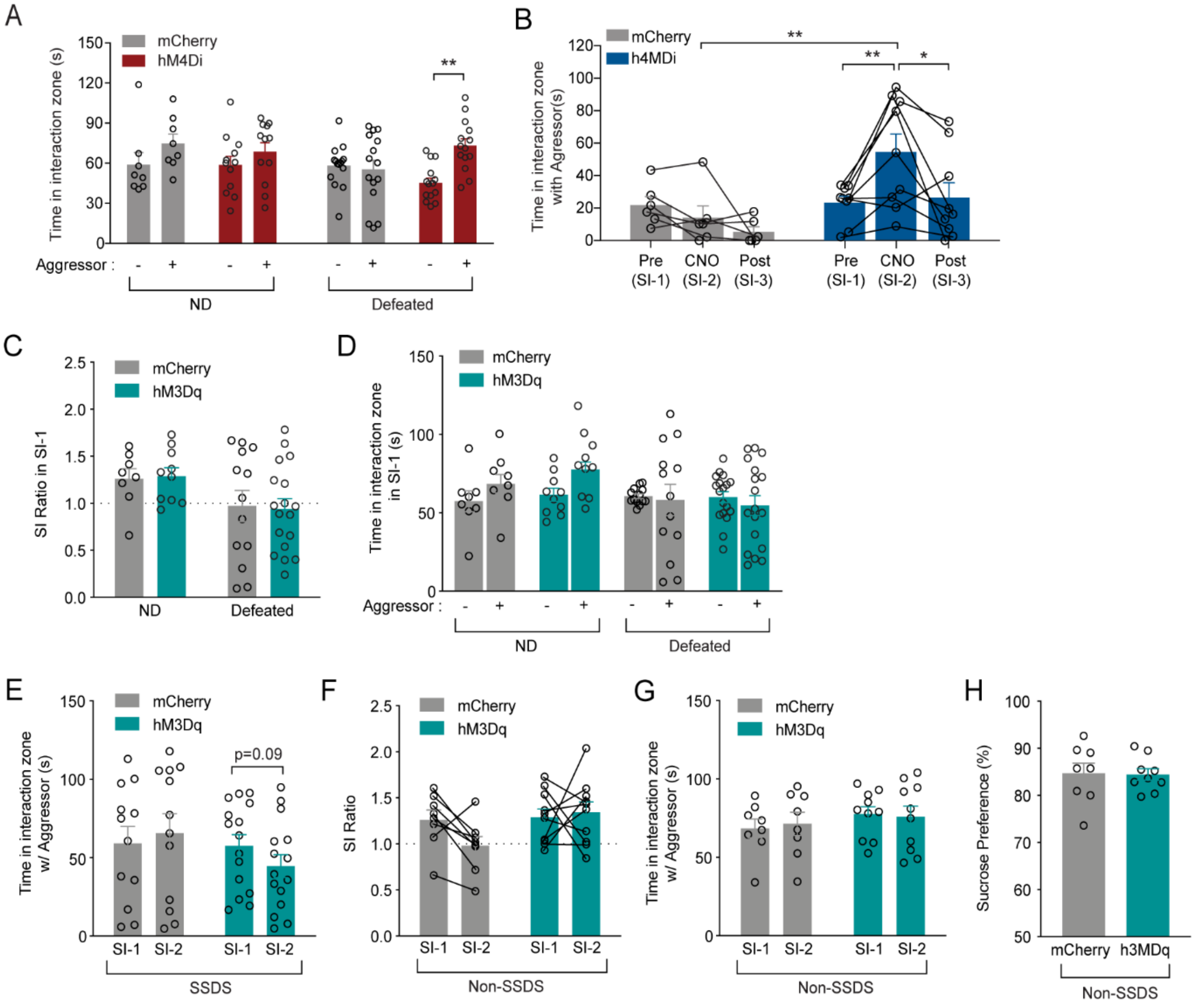

Supplementary Figure 2. Chemogenetic modulation of PV^{vDG} predisposes divergent behavioral outcome in response to social defeat stress. (A, B) Gi DREADD experiments. **(A)** Repeated CNO-treatment during CSDS resulted in increased time in the interaction zone with the aggressor compared to time in the empty interaction zone in hM4Di mice, while no difference was seen in mCherry controls (two-way repeated measures ANOVA, Non-defeat: mCherry (n=8) and hM4Di (n=12), Defeated: mCherry (n=15) and hM4Di (n=14)). **(B)** Acute CNO-treatment in susceptible mice resulted in increased time in the interaction zone with aggressor in hM4Di mice compared to mCherry controls and to treatment-free SI-tests (SI-1 and SI-3) (two-way repeated measures ANOVA, mCherry (n=6) and h4MDi (n=9)). **(C-H)** Gq DREADD experiments. **(C, D)** Acute activation during SSDS does not alter SI ratio (**C**) or time spent in the interaction zone (**D**) in hM3Dq or mCherry control mice (two-way repeated measures ANOVA, Non-defeat: mCherry (n=8) and h3MDq (n=10), Defeated: mCherry (n=13) and h3MDq (n=18)). **(E)** A trend of decreased time spent in the interaction zone in hM3Dq mice compared to mCherry controls was found after repeated injections of CNO post-SSDS (two-way repeated measures ANOVA, mCherry (n=12) and hM3Dq (n=15)). (**F, G**) Non-SSDS mice (social defeat-free) had no alterations in SI ratio (**F**) or time spent in the interaction zone (**G**) before or after repeated injections of CNO (two-way repeated measures ANOVA, mCherry (n=8) and hM3Dq (n=10)). (**H**) Repeated injections of CNO did not alter sucrose preference in non-SSDS mice (unpaired two-tailed t-test, mCherry (n=8) and hM3Dq (n=9)). Data are expressed as mean ± SEM, and individual data points are depicted. *Post-hoc* Bonferroni's multiple comparisons were used for ANOVA. *p<0.05, **p<0.01.

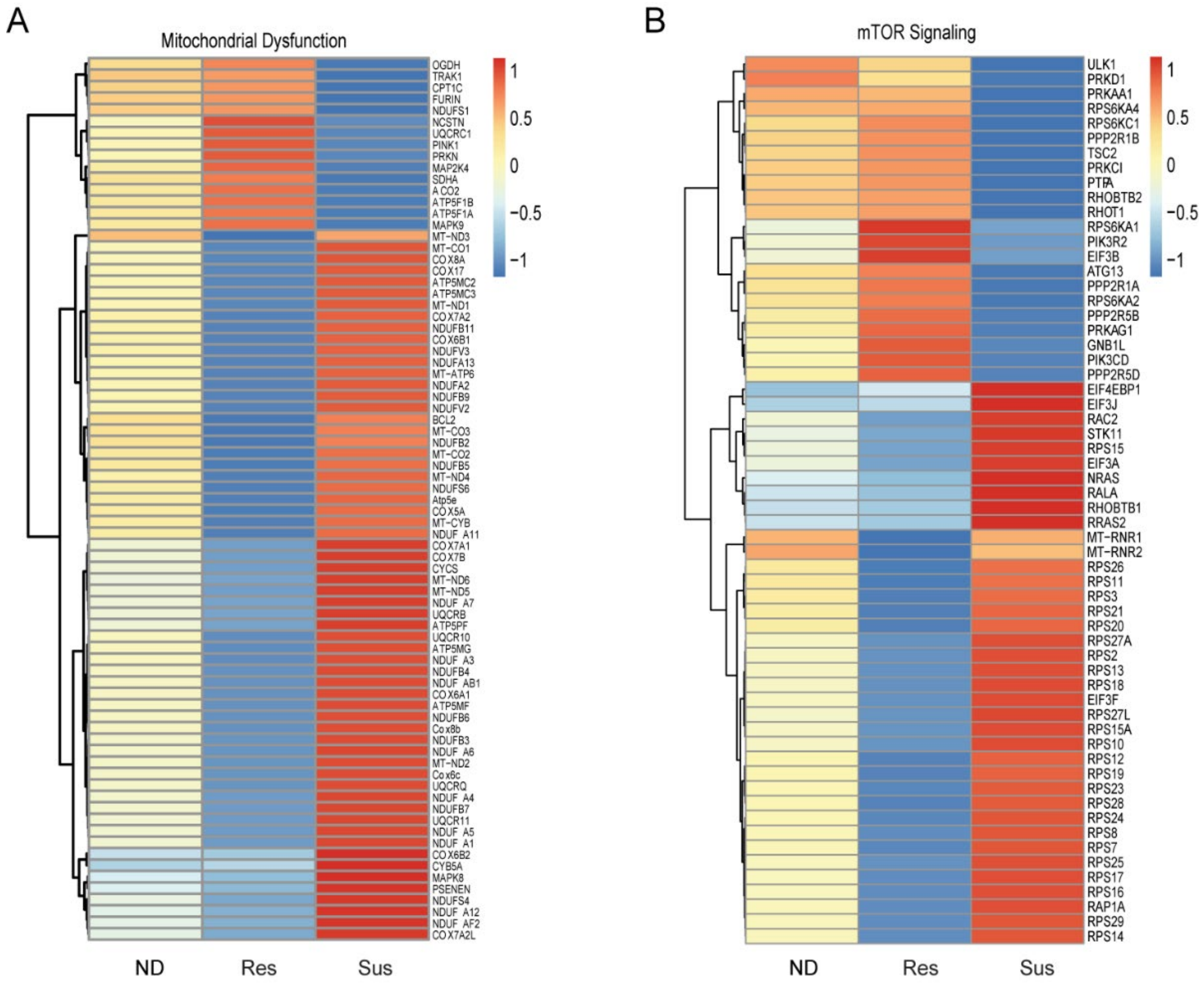

Supplementary Figure 3. Molecules associated with mitochondrial dysfunction and mTOR signaling are altered in hippocampal PV neurons in divergently behaving mouse groups after CSDS. (A, B) Heat maps of DEGs involved in mitochondrial dysfunction and mTOR signaling. Mean value of gene expression was used (Non-defeated (n=4), Res (n=4) and Sus (n=3)).

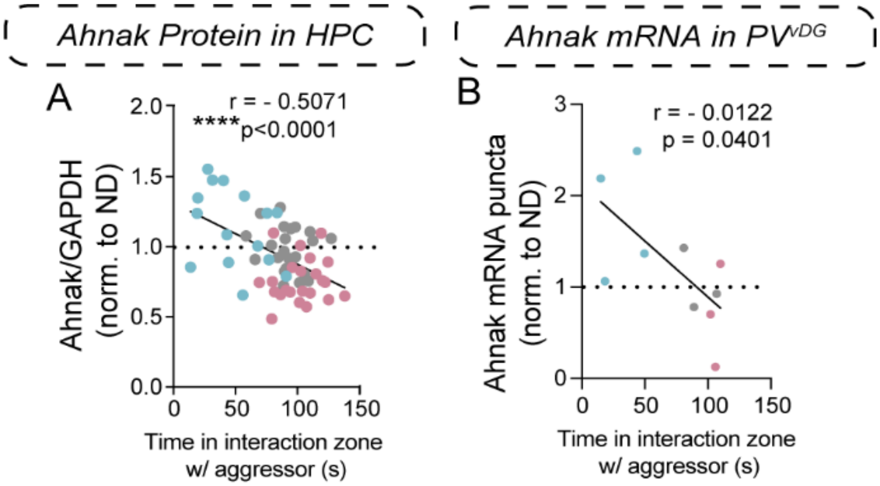

Supplementary Figure 4. Chronic stress induces alterations of Ahnak expression in the hippocampus. **(A)** Hippocampal Ahnak protein levels are inversely correlated with time spent in interaction zone with aggressor mouse (pearson *r* : *r* = -0.5071, *p*<0.0001, n=61 mice) (**B**) Ahnak mRNA RNAScope puncta are inversely correlated with time in interaction zone with aggressor (pearson *r* : *r* = -0.5071, *p*<0.0001, n=10 mice).

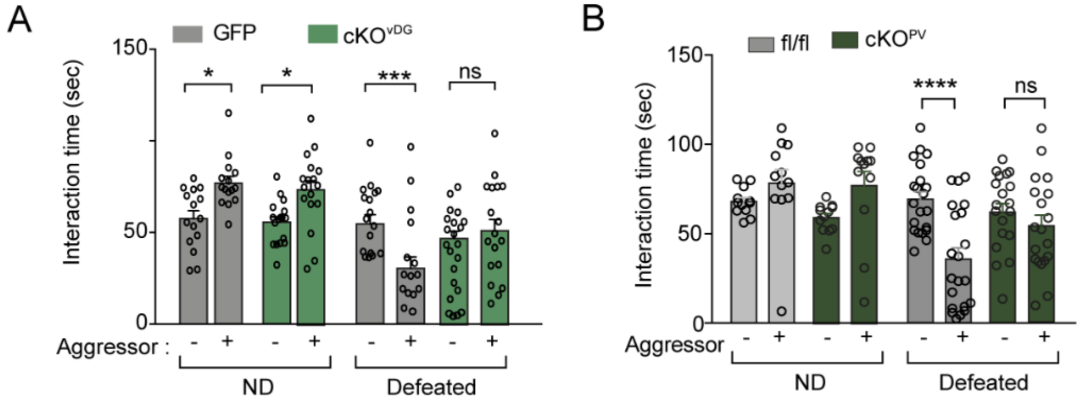

Supplementary Figure 5. Ahnak deletion in vDG or PV neurons confers resilience to CSDS. (**A**) In non-defeated conditions, both control and Ahnak cKO^{vDG} groups display higher amount of time spent in the interaction zone with an aggressor compared to the time without aggressor. After CSDS, control mice display significantly lower amount of time spent in the interaction zone with an aggressor compared to the time without aggressor, but Ahnak cKO^{vDG} mice display equal amounts of time in the interaction zone with or without an aggressor (two-way repeated measures ANOVA, Non-defeat: Control (n=15) and cKO^{vDG} (n=17), Defeated: Control (n=18) and KO^{vDG} (n=18)). (**B)** CSDS induces decreased interaction time during the aggressor session compared to empty-cage session in control group (floxed Ahnak mice, fl/fl), while the effect of CSDS on interaction time with an aggressor is abolished in Ahnak cKO^{PV} mice (two-way repeated measures ANOVA, Non-defeat: fl/fl (n=12) and cKO^{PV} (n=12), Defeated: fl/fl (n=21) and cKO^{PV} (n=19)). Data are expressed as mean ± SEM, and individual data points are depicted. *Post-hoc* Bonferroni's multiple comparisons were used for ANOVA. **p*<0.05, ****p*<0.001, *****p*<0.0001 and ns, nonsignificant.

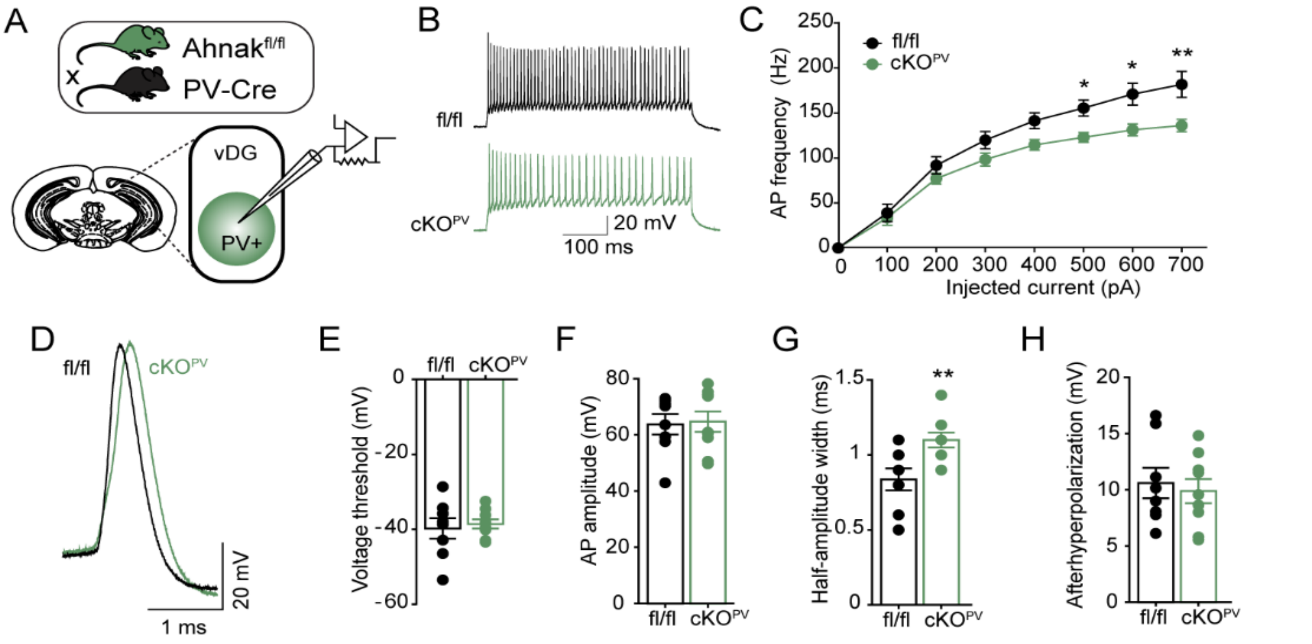

Supplementary Figure 6. The effect of PV neuron-selective Ahnak deletion on physiological properties of PV neurons in the vDG. (A) Schematic of whole-cell patch clamp. (**B**) Representative traces from whole-cell current-clamped PV neurons in the vDG of fl/fl and Ahnak cKO^{PV} mice showing the action potential (AP) firing of the cells in response to a 500 pA step of injected current. **(C)** AP frequency of PV neurons in the vDG is reduced in Ahnak cKO^{PV} mice at incremental steps of injected current (two-way ANOVA, fl/fl (n=10 neurons/4 mice) and cKO^{PV} (n=9 neurons/4 mice)). Data are expressed as mean ± SEM. *Post-hoc* Bonferroni's multiple comparisons were used for ANOVA. **(D)** Representative single APs in PV^{vDG} neurons from control (fl/fl, black) and Ahnak cKO^{PV} (green) mice. **(E-H)** AP properties of PV^{vDG} neurons in Ahnak cKO^{PV} and control mice. Ahnak cKO^{PV} does not influence the voltage threshold (**E**), AP amplitude (**F**), Afterhyperpolarization (**H**) but increases the half-amplitude width (**G**). Two-tailed unpaired *t*-test, 8 neurons/4 mice for control (fl/fl) and 9 neurons/mice for cKO^{PV} for each experiment). Data are expressed as mean ± SEM, and individual data points are depicted. *p<0.05, **p<0.01 and ns, nonsignificant.
