# Supplementary material for: Molecular and Cellular Adaptations in Hippocampal Parvalbumin Neurons Mediate Behavioral Responses to Chronic Social Stress
Source: Front Mol Neurosci. 2022 Jun 24;15:898851. doi: 10.3389/fnmol.2022.898851 (PMC9268684; doi:10.3389/fnmol.2022.898851)
Supplement: Supplementary file 2 [file Table_1.pdf]

Supplementary Table 1 Summary of statistical analysis (related to all Figures).

Figure		Experimental variables	Statistical test	Results	Post hoc tests
1	**B**	group (Non-defeat, Res, Sus)	one-way ANOVA	group: F(2,58)=40.82, *p*<0.0001	Bonferroni's multiple comparisons test<br>ND vs. Res: p =0.9763<br>ND vs. Sus: p<0.0001<br>Res vs. Sus: p<0.0001
	**F**	group (Cont, Res, Sus, Sus+Ket)	one-way ANOVA	group: F (3, 138) = 6.454, *p* = 0.0004	Bonferroni's multiple comparisons test<br>ND vs. Sus: p=0.0452<br>ND vs. Res: p>0.9999<br>ND vs. Sus+Ket: p>0.9999<br>Sus vs. Res: p=0.0131<br>Sus vs. Sus+Ket: p=0.0003<br>Res vs. Sus+Ket: p>0.9999
	**G**	group (Cont, Res, Sus, Sus+Ket)	one-way ANOVA	group: F (3, 202) = 5.632, *p* = 0.001	Bonferroni's multiple comparisons test<br>ND vs. Sus: p=0.0076<br>ND vs. Res: p>0.9999<br>ND vs. Sus+Ket: p>0.9999<br>Sus vs. Res: p=0.0499<br>Sus vs. Sus+Ket: p=0.0008<br>Res vs. Sus+Ket: p>0.9999
	**H**	group (Cont, Res, Sus, Sus+Ket)	one-way ANOVA	group: F (3, 130) = 4.673, *p* = 0.0039	Bonferroni's multiple comparisons test<br>ND vs. Sus: p>0.9999<br>ND vs. Res: p>0.9999<br>ND vs. Sus+Ket: p=0.0223<br>Sus vs. Res: p>0.9999<br>Sus vs. Sus+Ket: p=0.0064<br>Res vs. Sus+Ket: p=0.0131
	**I**	group (Cont, Res, Sus, Sus+Ket)	one-way ANOVA	group: F (3, 287) = 0.682, *p* = 0.5599	n/a
2	**D**	group (mCherry, hM4Di); treatment (non-defeat, defeated)	two-way ANOVA	group: F(1,45)=3.693, *p*=0.0610; treatment: F(1,45)=0.04982, *p*=0.8244; interaction, F(1,45)=10.23, *p*=0.0025	Bonferroni's multiple comparisons test<br>mCherry vs. hM4Di:<br>No Defeat: p=0.8300<br>Defeat: p=0.0004
	**F**	group (mCherry, hM4Di); treatment (non-defeat, defeated)	two-way ANOVA	group: F(1,48)=6.808, *p*=0.0121; treatment: F(1,48)=3.207, *p*=0.0796; interaction: F(1,48)=4.616, *p*=0.0368	Bonferroni's multiple comparisons test<br>mCherry vs. hM4Di:<br>No Defeat: p>0.9999<br>Defeat: p=0.0006

	**H**	time (Pre, CNO, Post); group (mCherry, hM4Di)	two-way repeated measures ANOVA	time: $F(2,26) = 1.805$, $p=0.1845$; group: $F(1,13)=2.834$, $p=0.1161$; interaction: $F(2,26)=4.945$, $p=0.0151$; subject: $F(13,26)=1.935$, $p=0.0737$	Bonferroni's multiple comparisons test mCherry vs. hM4Di:<br>Pre: p>0.9999<br>CNO: p=0.0052<br>Post: p>0.9999<br><br>Bonferroni's multiple comparisons test<br>mCherry:<br>Pre vs CNO: p>0.9999<br>Pre vs Post: p>0.9999<br>CNO vs Post: p>0.9999<br>hM4Di:<br>Pre vs CNO: p=0.0190<br>Pre vs Post: p>0.9999<br>CNO vs Post: p=0.0162
	**I**	hM4Di vs. mCherry	Two-tailed unpaired student's *t*-test	$t(14)=3.174$, $p=0.0068$	n/a
	**M**	time (SI-1, SI-2); group (mCherry, hM3Dq)	two-way repeated measures ANOVA	time: $F(1,25)=2.348$, $p=0.1380$; group: $F(1,25)=0.6389$, $p=0.4316$; interaction: $F(1,25)=2.756$, $p=0.1094$; subject: $F(25,25)=3.687$, $p=0.0009$	Bonferroni's multiple comparisons test<br>SI-1 vs SI-2<br>mCherry: p>0.9999<br>h3MDq: p = 0.0489
	**O**	hM3Dq vs. mCherry	two-tailed unpaired student's *t*-test	$t(25)=2.147$, $p=0.0416$	n/a
4	**C**	group (Non-defeat, Res, Sus)	one-way ANOVA	group: $F(2,59)=21.48$, $p<0.0001$	Bonferroni's multiple comparisons test<br>ND vs. Res: p =0.0002<br>ND vs. Sus: p=0.0266<br>Res vs. Sus: p<0.0001
	**D**	SI Ratio vs. Ahnak protein	Pearson's Correlation	R2=0.1471, $p=0.0008$	n/a
	**F**	group (Non-defeat, Res, Sus)	Per Cell: Krusal-	Per Cell:<br>Krusal-Wallis statistic: 38.89, p<0.0001	Dunn's multiple comparisons test<br>ND vs. Res: p =0.0010<br>ND vs. Sus: p=0.0092

			Wallis test		Res vs. Sus: p<0.0001
	**G**	SI Ratio vs. normalized Ahnak puncta per cell	Pearson's Correlation	R2=0.4911, *p*=0.0240	n/a
5	**C**	group (GFP, cKO^{vDG}); treatment (non-defeat, defeated)	two-way ANOVA	group: F(1,64)=20.48, *p*<0.0001; treatment: F(1,64)=3.190, *p*=0.0788; interaction, F(1,64)=5.543 *p*=0.0216	Bonferroni's multiple comparisons test<br>GFP vs. cKO^{vDG}<br>ND: p >0.9999<br>Defeated: p=0.0072
	**E**	group (GFP, cKO^{vDG}); treatment (non-defeat, defeated)	two-way ANOVA	group: F(1,27)=7.450, *p*=0.0110; treatment: F(1,27)=3.377, *p*=0.0771, interaction, F(1,27)=2.126, *p*=0.1564	Bonferroni's multiple comparisons test<br>GFP vs. cKO^{vDG}<br>ND: p >0.9999<br>Defeated: p=0.0268
	**H**	group (fl/fl, cKO^{PV}); treatment (non-defeat, defeated)	two-way ANOVA	group: F(1,60)=4.796, *p*=0.0324; treatment: F(1,60)=12.92, *p*=0.0007; interaction, F(1,60)=1.289, *p*=0.2608	Bonferroni's multiple comparisons test<br>fl/fl vs. cKO^{PV}<br>ND: p >0.9999<br>Defeated: p=0.0174
	**J**	group (fl/fl, cKO^{PV}); treatment (non-defeat, defeated)	two-way ANOVA	group: F(1,43)=9.779, *p*=0.0032; treatment: F(1,43)=34.76, *p*<0.0001; interaction, F(1,43)=1.239, *p*=0.2719	Bonferroni's multiple comparisons test<br>fl/fl vs. cKO^{PV}<br>ND: p =0.3109<br>Defeated: p=0.0101
S1		time (absence, presence of aggressor); group (non-defeat, Res, Sus)	two-way repeated measures ANOVA	time: F(1,58) = 0.5697, *p*=0.4534; group: F(2,58) = 11.10, *p*<0.0001; interaction: F(2,58)=42.24, *p*<0.0001; subject: F(58,58)=1.590, *p*=0.0401	Bonferroni's multiple comparisons test<br>Empty vs. Aggressor<br>ND: p<0.0001<br>Res: p<0.0001<br>Sus: p<0.0001
S2	**A**	time (absence, presence of aggressor); group (non-defeat mCherry, non-defeat hM4Di, defeated mCherry, defeated hM4Di)	two-way repeated measures ANOVA	time: F(1,45)=9.803, *p*=0.0031; group: F(3,45)=0.9129, *p*=0.4423; interaction, F(3,45)=3.042, *p*=0.0384; subject: F(45,45)=1.274, *p*=0.2101.	Bonferroni's multiple comparisons test<br>Empty vs. Aggressor<br>ND-mCherry: p=0.4405<br>ND-hM4Di: p=0.8719<br>Defeated-mCherry: p>0.9999<br>Defeated-hM4Di: p=0.0019
	**B**	time (SI-1, SI-2, SI-3); group (mCherry, hM4Di)	two-way repeated measures ANOVA	time: F(2,26)=4.214, *p*=0.0260; group: F(1,13)=5.703, *p*=0.0328; interaction, F(2,26)=4.945, *p*=0.0151; subject: F(31,31)=4.620, *p*<0.0001	Bonferroni's multiple comparisons test<br>mCherry vs. hM4Di<br>Pre: p>0.9999<br>CNO: p=0.0034<br>Post: p = 0.2192

					Bonferroni's multiple comparisons test<br>mCherry-<br>Pre vs. CNO: p=0.9689<br>Pre vs. Post: p=0.4947<br>CNO vs. Post: p=0.9465<br>hM4Di-<br>Pre vs. CNO: p=0.0042<br>Pre vs. Post: p=0.9993<br>CNO vs. Post: p=0.0113
	**C**	group (mCherry, hM3Dq); treatment (non-defeat, defeated);	two-way ANOVA	group: F(1,45)=0.0002492, *p*=0.9875; treatment: F(1,45)=5.623, *p*=0.0221; interaction, F(1,45)=0.0474, *p*=0.8287	n/a
	**D**	time (aggressor, no agresor); group (non-defeat mCherry, non-defeat hM43q, defeated mCherry, defeated hM3Dq);	two-way repeated measures ANOVA	time: F(1,45)=1.465, *p*=0.2324; group: F(3,45)=1.310, *p*=0.2830; interaction, F(3,45)=1.752, *p*=0.1700; subject, F(45,45)=1.445, *p*=0.1105	n/a
	**E**	Time (SI-1, SI-2); group (mCherry, hM3Dq)	two-way repeated measures ANOVA	time: F(1,25)=0.4731, *p*=0.4979, group: F(1,25)=0.8439, *p*=0.3671; interaction: F(1,25)=4.408, *p*=0.0460, subject: F(25,25)=6.997, *p*<0.0001	Bonferroni's multiple comparisons test<br>SI-1 vs SI-2<br>mCherry: p=0.7054<br>hM3Dq: p = 0.0938
	**F**	Time (SI-1, SI-2); group (mCherry, hM3Dq)	two-way repeated measures ANOVA	time: F(1,16)=1.172, *p*=0.2951; group: F(1,16)=3.803; *p*=0.0689; interaction, F(1,16) = 2.534, *p* = 0.1310; F(16,16)=0.88997, *p*=0.5824	n/a
	**G**	Time (SI-1, SI-2); group (mCherry, hM3Dq)	two-way repeated measures ANOVA	time: F(1,16)=0.008782, *p*=0.9265, group: F(1,16)=1.111, *p*=0.3074; interaction: F(1,16)=0.1347, *p*=0.7184; subject: F(16,16)=0.9884, *p*=0.5092	n/a
	**H**	hM3Dq vs. mCherry	two-tailed unpaired student's *t*-test	t(15)=0.09129, *p*=0.9285	n/a

S4	**A**	Time in interaction zone w/ Agg. Ahnak protein	Pearson's Correlation	R2=0.2572, *p*<0.0001	n/a
	**B**	Time in Agg Zone vs. normalized Ahnak puncta per cell	Pearson's Correlation	R2=0.4282 *p*=0.0401	n/a
S5	**A**	time (aggressor, no aggressor); group (non-defeat GFP, non-defeat cKO-vDG, defeated GFP, defeated cKO-vDG);	two-way repeated measures ANOVA	time: F(1,64)=0.2.057, *p*=0.1564; group: F(3,64)= 9.806, *p*<0.0001; interaction, F(3,64)=11.19, *p*<0.0001; subject, F(64,64)=1.566, *p*=0.0375	Bonferroni's multiple comparisons test<br>Empty vs. Aggressor<br>ND-GFP: p=0.0167<br>ND-cKO^{vDG}: p=0.0160<br>Defeated GFP: p=0.0005<br>Defeated cKO^{vDG}: p=0.9245
	**B**	time (aggressor, no agressor); group (non-defeat fl/fl, non-defeat cKO^{PV}, defeated fl/fl, defeated cKO^{PV});	two-way repeated measures ANOVA	time: F(1,60)=0.8963, *p*=0.3476; group: F(3,60)= 4.853, *p*=0.0043; interaction, F(3,60)=9.608, *p*<0.0001; subject, F(60,60)=1.291, *p*=0.1625	Bonferroni's multiple comparisons test<br>Empty vs. Aggressor<br>ND-fl/fl: p=0.6562<br>ND-cKO^{PV}: p=0.1557<br>Defeated-fl/fl: p<0.0001<br>Defeated-cKO^{PV}: p=0.5232
S6	**C**	group (fl/fl, cKO^{PV}); injected current	two-way ANOVA	firing at increased injected current (time): F (7, 122) = 97.53, *P* < 0.0001;<br>firing at the same injected current (group): F (1, 122) = 31.68, *P* < 0.0001;<br>Interaction: F (7, 122) = 1.791, *P* = 0.0949	Bonferroni's multiple comparison; fl/fl vs. cKO^{PV}:<br>0 pA: p>0.9999<br>100 pA: p>0.9999<br>200 pA: p>0.9999<br>300 pA: p=0.4075<br>400 pA: p>0.1245<br>500 pA: p=0.0277<br>600 pA: p=0.0367<br>700 pA: p=0.0095
	**E**	group (fl/fl, cKO^{PV})	unpaired two-tailed student's *t* test	*p* = 0.6777, t(15)=0.4238	n/a

	**F**	group (fl/fl, cKO^{PV})	unpaired two-tailed student's *t* test	p = 0.8552, t(15)=0.1856	n/a
	**G**	group (fl/fl, cKO^{PV})	unpaired two-tailed student's *t* test	p = 0.0085, t(15)=3.024	n/a
	**H**	group (fl/fl, cKO^{PV})	unpaired two-tailed student's *t* test	p = 0.6841, t(15)=0.4148	n/a
